# Meta-analytic pooling of intraclass correlation coefficient estimates

**DOI:** 10.1017/rsm.2026.10077

**Published:** 2026-03-30

**Authors:** Bethany H. Bhat, S. Natasha Beretvas

**Affiliations:** 1 Department of Psychiatry and Neuropsychology, Maastricht University, Maastricht, Netherlands; 2 Educational Psychology, The University of Texas at Austin, USA

**Keywords:** cluster, intraclass correlation coefficient, meta-analysis, power analysis, reliability

## Abstract

Intraclass correlation coefficient (ICC) estimates are necessary for several statistical techniques. Researchers need accurate ICC estimates when conducting prospective power analyses for clustered data scenarios. In addition, meta-analysts require reasonable ICC values when adjusting effect size estimates to account for clustered primary study data or to correct for psychometric artifacts when using the ICC as a reliability measure. The validity of these analyses hinges on the accuracy of the ICC estimate. Beyond these secondary analyses, ICC estimates have been used as the focal outcome of meta-analysis itself to obtain a pooled measure of agreement, reliability, or the influence of a cluster’s effect. This study evaluates how well meta-analytically pooled ICC estimates recover the population ICC parameter value when using different ICC variance formulas as the inverse variance weights used in the pooling. We found that the variance formula that uses a normalizing transformation performs best across most conditions.

## Highlights

### What is already known?

- Accurate intraclass correlation coefficient (ICC) estimates are needed for power analyses and meta-analytic adjustments to effect sizes from clustered data.
- The ICC does not have a known sampling distribution shape, nor have a known sampling variance.
- Not all meta-analytic pooling techniques are robust when variance formulas are mis-specified or when the effect size estimate’s sampling distribution is asymmetric.

### What is new?

- An empirical evaluation of different ICC variance formulas used as weights in meta-analytic pooling.
- Identification of the Fisher transformation-based formula as the most accurate weighting approach across varied conditions.

### Potential impact for RSM readers

- Provides guidance on selecting variance formulas when pooling ICC estimates to be used in power analyses and effect size corrections for clustered data.

## 1 Introduction

The intraclass (or intracluster) correlation coefficient (ICC)^1^ ^–^ ^3^ captures the degree of clustering (or similarity) in the data. ICC estimates are necessary for several statistical techniques for handling clustered data arising from contexts, such as cluster-randomized trials (CRTs) or studies assessing the consistency of measurements across subjects. The CRT is an experimental design that involves randomly assigning entire clusters of individuals (such as classrooms of students or hospitals of patients) to treatment conditions.^4^ ^–^ ^7^ In meta-analysis, the estimated ICC is necessary to adjust effect sizes from studies with clustered data.^8^ ^–^ ^11^ However, primary studies with clustered data do not always report ICC estimates, requiring meta-analysts to impute ICC values from other studies with similar design characteristics. Similarly, in an a priori power analysis, reasonable ICC estimates are needed if the experimental design of the planned study involves clustered data such as a CRT.

ICC estimates are necessary when clustered data are encountered both in meta-analysis and a priori power analysis because the standard error (SE) of a treatment effect in clustered data is inflated by the variance inflation factor, which is itself a function of the ICC. Even if the magnitude of the ICC estimate (and thus the degree of cluster-dependence) is small, if the average size of a cluster is large, then the ICC estimate has a major effect on the SE of the treatment effect.^12^ The accuracy of both types of clustered data analyses depends on the accuracy of the ICC estimates used. Because raw data are not available for prospective power analyses or meta-analyses, researchers typically have to impute reasonable values from ICC estimates reported in related prior studies.

To address the need for accurate ICC values for clustered data, large secondary databases of design parameters have been used to offer reasonable ICC values for a set of research designs, outcomes, and population groups.^13^ ^–^ ^16^ Unfortunately, such databases and, thus, ICC values are not comprehensive for all designs, outcomes, and populations. In addition, even if the ICCs captured in those datasets were based on very similar studies, the sampling distribution of ICC estimates is unknown, making it unclear how best to pool together ICC estimates from such databases.

Beyond the context of clustered individuals’ data, the ICC metric is also used as a measure of reliability for multiple measures of a construct. An ICC in this context is the proportion of the variance due to the objects of a measurement.^3^ ^,^ ^17^ There are many types of ICCs in this context (see McGraw and Wong^3^ for a review), but for this article, we are interested in the ICC from a one-way random effects model, in which unordered measurement observations are nested within objects (e.g., participants). For example, a sample of participants is each rated by the same set of raters, and the ICC provides a measure of inter-rater reliability. Alternatively, multiple measurements are taken for a group of participants across multiple time points as a measure of test–retest reliability.^17^ Some meta-analysts correct the standardized mean difference (SMD) effect sizes for measurement artifacts. When unaccounted for, measurement error in the dependent variable can impact the SMD effect size by increasing the magnitude of the within-group variance used to calculate each primary study’s SMD estimate. When the SMD estimates are not corrected for measurement error, the pooled SMD estimate can be under-estimated, heterogeneity in effect size estimates can be inflated, and there can be other negative implications, including erroneous inferences made about moderator effects and publication bias.^18^ The ICC, as a measure of inter-rater reliability, is among the reliability measures that some meta-analysts use to correct the SMD for rater error.^18^ Thus, the ICC has multiple important uses.

Several methods have been proposed to provide ICC values for secondary analyses. Some applied researchers have taken the arithmetic mean or median of a set of ICC estimates from a similar population context.^19^ ^,^ ^20^ Others have constructed confidence intervals for the ICC parameter and proposed that applied researchers use the upper limit as a conservative estimate of the ICC.^21^ In the case of power analysis, this method would produce a sample size larger than needed and could result in wasted resources.^21^ Finally, some have applied Bayesian methods to create a posterior distribution for the ICC estimates.^22^ ^–^ ^24^ These methods have relied on strong assumptions about the distribution of ICC estimates and its moments. In addition, simple averages of ICCs do not capture the differing precisions of the ICC estimates. ICC estimates based on larger sample sizes will be more precise than those based on smaller sample sizes. More weight should be assigned to more precise ICC estimates when calculating a pooled ICC.

Thus, another alternative for imputing a reasonable ICC estimate can involve the use of principled meta-analytic methods that are more typically used to pool effect sizes. When pooling effect sizes, a meta-analyst can calculate a weighted average effect size where weights are a direct function of precision. Most typically, the inverse of the effect size’s variance is used as the effect size’s weight, such that the more precise the estimate, the smaller its SE and the more weight it is afforded.^25^ Similarly, a researcher could pool ICC estimates from different studies’ datasets and assign more weight in the pooling to more precise ICC estimates. However, accurate use of meta-analytic pooling requires knowledge of the shape and variance of the sampling distribution of the effect size of interest (here, of ICC estimates). While many different formulas for the variance of an ICC estimate have been proposed,^1^ ^,^ ^2^ ^,^ ^26^ ^–^ ^29^ it is unclear which formulation of the variance of ICC estimates might offer the best weight formulation when pooling ICC estimates to obtain an estimate of the population ICC. Furthermore, ICC estimates have already been used as an outcome in meta-analyses, particularly in psychology, education, and the health sciences,^15^ ^,^ ^30^ ^–^ ^33^ although the sampling variance formula used in the weights and the meta-analysis model has varied across these studies, which further justifies the need for a simulation study investigating the performance.

The aim of this study is to compare methods for quantitatively synthesizing ICC values to obtain the best pooled ICC estimate. There are several choices for how to pool effect sizes (for this study, ICCs) using meta-analysis. Two options for random effects pooling of effect sizes include method of moments estimation in the robust variance estimation (RVE) framework^34^ and restricted maximum likelihood estimation (REML).^35^ RVE is robust to the choice of distributional form of the effect size being pooled and should be less impacted by the effect size weights used in the meta-analytic model.^34^ Because the distributional form and sampling variance of ICC estimates are unknown, a meta-analytic technique, such as RVE, seems well matched for meta-analysis of ICC estimates. For comparison, we also evaluated REML estimation with random effects pooling, as it remains a commonly used estimation procedure in meta-analysis. In addition to comparing estimation procedures, we also investigated how well different proposed formulas for the variance of the ICC sampling distribution performed in the inverse variance weights used when pooling ICC estimates. This study is intended to provide guidelines for obtaining unbiased pooled ICC estimates that ultimately can be used in multiple scenarios, including a priori power analyses and meta-analysis of effect size estimates for scenarios with clustered individual or measurement data.

### 1.1 Why do we need estimates for ICCs for secondary analyses?

One goal of this study is to evaluate the synthesis of ICC sample estimates in order to obtain an accurate estimate of an ICC for a secondary analysis when the raw data are not available. There are two common contexts in which this is needed: (1) prospective power analyses when designing a CRT and (2) meta-analysis of effect sizes that need to be corrected for clustering or psychometric artifacts.

#### 1.1.1 Power analysis

When designing an experiment, it is necessary to determine the sample size needed to achieve sufficient statistical power to detect a pre-specified minimal treatment effect. A prospective power analysis for two-level clustered data requires specifying the desired power, the appropriate α -level, the expected minimum effect size, a plausible value for the ICC and either the total sample size (when determining the number of clusters needed) or the total number of clusters (when determining the total sample size).^36^ ^–^ ^38^ The sample size formulas that do not account for clustering will underestimate the number of required individuals in clustered data.

The variance inflation factor (VIF) accounts for clustering, and the power to detect the true effect size is dependent on the ICC estimate. VIF is equal to: VIF=1+(n−1)ρ , where ρ is the ICC and *n* is the average number of within-cluster units. The VIF is a multiplicative factor that quantifies the increase in variance due to randomizing clusters into treatment conditions. This shows that if either the ρ , *n*, or both ρ and *n* are larger then this results in a large increase in the variance of the treatment effect estimate. Because CRTs have a larger variance for the treatment effect and have fewer degrees of freedom than designs that randomize students,^12^ CRTs also have relatively less power. So if the number of clusters in a study is small, the power analysis is highly dependent on the ρ estimate.

It would be ideal to know the ICC prior to conducting a power analysis; however, because it is not possible to know the ICC before collecting the data, researchers must impute an expected ICC from previously conducted studies that are similar. Mis-specifying the ICC in a power analysis can lead to a drastic miscalculation of the sample size necessary to achieve a desired level of power. If the estimate for ρ used in the prospective power analysis’ sample size calculation is lower than the true ICC value, the necessary sample size will be underestimated, and the study will be under-powered. Alternatively, using a conservative ρ estimate (i.e., larger than the true ICC) in a prospective power analysis will inflate the sample size used, thereby unnecessarily wasting resources in the actual study.

Furthermore, while it is advisable to do a sensitivity analysis for the assumed ICC when conducting power analyses, a more accurate ICC value provides a necessarily better starting point for potential sensitivity analyses. Considering the number of design parameters that also need to be considered in a power analysis, the use of the optimal ICC value can assist applied researchers in the initial planning. Ultimately, a researcher must decide on a target sample size, and a suitable ICC value helps in that determination.

#### 1.1.2 Correcting effect sizes for artifacts in meta-analysis

*Correcting for clustering:* Meta-analysis offers another scenario in which accurate ICC estimation can be critical. Meta-analysis is a collection of statistical procedures that evaluate and synthesize findings from a comprehensive set of primary studies on a similar topic. Meta-analysts summarizing treatment effects across relevant primary studies may need to capture effect sizes from studies with varying experimental designs. These effect sizes must first be standardized using a common metric before they can be included in the meta-analysis. For example, if the treatment effect at the individual level is of interest to the meta-analyst, then effect sizes from CRTs must be adjusted to reflect individual-level effects using the ICC.^8^ ^,^ ^9^ Often, primary studies do not report an ICC value, nor do they provide sufficient information for a meta-analyst to calculate it manually. Thus, as with power analyses, researchers must impute ICC estimates prior to making the adjustments necessary to include a primary study’s clustered data’s effect size estimates and associated variances. For two-level data, such methods were first introduced in Hedges^8^ and later corrected in *Supplement to the What Works Clearninghouse Procedures Handbook, Version 4.1.* ^39^ For three-level data using MLM, the formulas for effect sizes adjusted for clustering are presented in Hedges.^9^

If a meta-analyst wanted to calculate Hedges’ *g* for a CRT with one level of clustering (i.e., a two-level design), they would use the following formula:

(1.1) $$\begin{array}{r} g=\frac{\omega ({\overset{―}{Y}}_{T}-{\overset{―}{Y}}_{C})}{s_{p}}\sqrt{1-\frac{2(n-1)\hat{\rho }}{N-2}}, \end{array}$$

where $\hat{\rho }$ is the ICC estimate, *N* is the total number of level-1 units, *n* is the average cluster size, $s_{p}$ is the pooled within-group standard deviation, ${\overset{―}{Y}}_{T}$ is the mean of the individuals in the treatment group, ${\overset{―}{Y}}_{C}$ is the mean of the individuals in the control group, ω is the small sample size adjustment equal to 1+3/(4df−1) , and $\sqrt{1-\frac{2(n-1)\hat{\rho }}{N-2}}$ corrects the effect size for upward bias when $\hat{\rho }$ is greater than 0 . As can be seen from Equation (1.1), the cluster-adjusted effect size is dependent on the $\hat{\rho }$ estimate. The df is the degrees of freedom for a two-level model and is found using the following expression^11^ ^,^ ^40^:

$$\begin{array}{r} df=\frac{[(N-2)-2(n-1)\hat{\rho }{]}^{2}}{\left(N-2)(1-\hat{\rho }{)}^{2}+n(N-2n){\hat{\rho }}^{2}+2(N-2n)\hat{\rho }(1-\hat{\rho }\right)}. \end{array}$$

The equation for the cluster-assignment effect size SE is as follows:

$$\begin{array}{r} \begin{array}{rl} se & =\omega \;\times \\ & \sqrt{\left[\frac{N}{n_{T}n_{C}}\right]\left(1+\left(n-1\right)\hat{\rho }\right)+g^{2}\frac{\left(N-2)(1-\hat{\rho }{)}^{2}+n\left(N-2n\right){\hat{\rho }}^{2}+2\left(N-2n\right)\hat{\rho }(1-\hat{\rho }\right)}{2[(N-2)-2\left(n-1\right)\hat{\rho }{]}^{2}}}, \end{array} \end{array}$$

where $n_{T}$ is the number of individuals in the treatment group and $n_{C}$ is the number of individuals in the control group. This SE is used as weights (by squaring the SE to obtain the associated sampling variance for the adjusted effect size and then taking the inverse of the variance) when combining effect size estimates across studies of different experimental designs.^11^ ^,^ ^40^ These equations highlight the importance of the ICC value used in terms of its contribution to both the adjusted SMD effect size for clustered data and its sampling variance.

The Cochrane Handbook for Systematic Reviews of Interventions^10^ has an alternative approach to accounting for CRTs (or even individually randomized trials with clustering) in a meta-analysis. They suggest multiplying the SE of the effect size estimate (continuous or dichotomous) by the square root of the VIF (or as they refer to it as the design effect).^10^

*Correcting effect sizes for psychometric artifacts:* While there are different types of reliability coefficients that account for different sources of measurement error variance, the ICC as an inter-rater reliability coefficient can be used to correct SMD effects for measurement error in observer ratings.^18^ One way that this artifact correction is obtained is by calculating the ICC as a reliability coefficient for each group (e.g., treatment and control groups). The pooled sample-size-weighted average of the ICC estimates ( $\overset{―}{\hat{\rho }}$ ) is then calculated and used to correct the observed SMD ( $d_{obs})$ :

$$\begin{array}{r} d_{c}=\frac{d_{obs}}{\sqrt{\overset{―}{\hat{\rho }}}}, \end{array}$$

where $d_{c}$ is the corrected SMD.^18^

## 2 Literature review

As emphasized, there are multiple scenarios in which accurate ICC estimates are needed. One potential method for obtaining an accurate ICC estimate consists of pooling reported values from related prior studies. However, there is a lack of consensus about how best to pool ICCs. In the following few sections, we review the research on methodological considerations when determining the best method for pooling ICC estimates. First, we provide a very brief overview of clustered data analysis, which serves as the framework for understanding ICCs and provides a formal definition of the ICC. Next, we explain the importance of good ICC estimates and review past methods for imputing them when conducting power analyses and meta-analyses. We describe what is known about the sampling distribution of ICC estimates, including the associated variance formulations. Last, we summarize the meta-analytic methods relevant to pooling ICC estimates.

### 2.1 Analyzing clustered data

While several analytic methods have been developed to handle clustered data,^28^ ^,^ ^41^ ^,^ ^42^ multilevel modeling (MLM; also referred to as mixed-effects, random-effects, hierarchical linear, and random-coefficients modeling^4^) is a common modeling technique used in applied social science research. When the functional form of an MLM model is correctly specified for the data structure, MLM has been shown to result in more precise SEs.^42^ MLM models cluster dependence by portioning the overall variance in an outcome into within-cluster and between-cluster components. A common applied data scenario entails lower-order units (like students) that are clustered within higher-order units (like schools). In this study, we focus on two-level data in which only the lower-level units are nested within only one level of higher-level units. Future research can explore scenarios with additional levels and further complications in clustered data.

In the MLM framework, both the between- and the within-cluster variation must be specified in the model.^4^ For example, in a dataset with students nested within schools, the nested structure can be modeled using a two-level random-effects model as follows:

(2.1) $$\begin{array}{r} \begin{array}{rl} Level\;1:Y_{ij} & =\beta_{0j}+e_{ij}\\ Level\;2:\beta_{0j} & =\gamma_{00}+u_{0j}, \end{array} \end{array}$$

where $Y_{ij}$ is the outcome score for student *i* in school *j*, $\gamma_{00}$ is the grand mean of the outcome $Y_{ij}$ , $u_{0j}$ is the random effect for school *j*, and $e_{ij}$ is the level-1 residual that varies around school *j*’s mean, $\beta_{0j}$ . Both residual terms, $u_{0j}$ and $e_{ij}$ , are typically assumed to follow independent normal distributions, each with a mean of zero and variances of $\sigma_{u}^{2}$ and $\sigma_{e}^{2}$ , respectively. In the above specification, we assume errors from different clusters are independent, while errors within a cluster are modeled as correlated. The correlation is commonly assumed to be the same for each pair of outcomes within and across clusters. More complex specifications are available, but for the purpose of this study, we will maintain these assumptions.

### 2.2 Intraclass correlation coefficient

In the MLM framework, the ICC is used to summarize the degree of dependence in the data by capturing the proportion of total variance in the outcome that is attributable to between-cluster variation. For MLMs, the variance within clusters is typically assumed to be the same across clusters. Formally, the equation for the ICC, denoted as ρ , from a two-level unconditional model, a model with no predictors at either level as depicted in Equation (2.1), is as follows:

(2.2) $$\begin{array}{r} \rho =\frac{\sigma_{u}^{2}}{\sigma_{u}^{2}+\sigma_{e}^{2}}, \end{array}$$

where ρ equals the level-2 variance divided by the level-2 variance plus the level-1 variance, or total variance. The ICC is non-negative, ρ≥0 , because variance components are non-negative. Higher ICC values imply that there is a stronger clustering effect, and an ICC value of zero implies that there is no clustering dependence. Another application of the ICC is as a reliability estimate for the degree of absolute agreement among measurements from a one-way random effects model.^3^ ^,^ ^17^ ^,^ ^43^ Although typically, the ICC in this context was computed in the ANOVA framework,^3^ it can also be estimated using the MLM framework.^17^ For example, in this context, the level-2 units could be subjects, and each subject is measured by the same instruments (e.g., raters or repeated measurements), which would be the level-1 units.

The ICC is typically estimated using an unconditional model as shown in Equation (2.1).^8^ ^,^ ^14^ That being said, however, it is possible to estimate a conditional ICC that incorporates the change in the variance explained by including covariates in the MLM. However, in this study, we focused on unconditional ICCs because that is what is typically used as the ICC value in meta-analysis and a priori power analyses for clustered data.^40^ Use of unconditional ICCs offers a starting point for this line of research. Future research can begin to consider how best to synthesize conditional ICCs.

The magnitude of an ICC estimate in the context of clustered data is dependent on the study’s experimental design, outcome measure, and population. Despite the full range of ICC values being between 0 and 1, ICC values estimated from real data will have a restricted range depending on the context. For example, the ICC estimates reported by Hedges and Hedberg^13^ range from 0.045 to 0.271 in educational research settings. Yet ICC values as low as 0.05 and 0.10 can result in severe type-I error biases if clustering is ignored in the associated analyses.^44^ ^,^ ^45^ In the context of measurement and reliability, typical ICC values can be much higher, with a common categorization of ICC values of less than 0.5, 0.5–0.75, 0.75–0.9, and greater than 0.9 being generally interpreted as indicating poor, moderate, good, and excellent reliability, respectively.^46^ Essentially, for a measurement to be considered reliable in this context, the level-1 variance needs to be much smaller compared to the level-2 variance. While these classifications are not universal and the context of the study should always be considered, we mention this categorization to contextualize the expected magnitude for ICC estimates when used as a reliability measure.

### 2.3 Review of past methods to handle missing ICC values

In the following section, we review the methods that have been proposed when an ICC estimate is needed for a prospective power analysis or a meta-analysis of clustered data. These methods are not evaluated in this study due to the limitations that we will present in this section. However, these methods help inform some of the problems that should be considered when tackling this methodological dilemma.

#### 2.3.1 Single-value imputation methods

As stated earlier, an inaccurate ICC value can have negative consequences on the result of either a power analysis or a meta-analysis of CRTs. One possible method used to estimate an ICC in the case of a prospective power analysis is to run a pilot study^47^ entailing a much smaller dataset than the proposed full study. However, a pilot study will result in imprecise level-1 and level-2 variance estimates and thus also in the resulting ICC estimate based on the small pilot study. In addition, funding and time constraints further limit the practicality of running a pilot study.

One option is to impute an ICC value from a prior study with a similar design. In educational and medical research, several investigators have compiled typical design parameters, including sample sizes and associated ICC values, for multilevel experimental designs from data collected at the cluster unit or national level for single-value imputation purposes.^13^ ^,^ ^15^ ^,^ ^48^ These authors have found that ICCs varied depending on a number of design characteristics, including, for example, the outcome measure, age, and socioeconomic status.

When researchers are unable to find studies with sufficiently similar design parameters, they employ ad hoc procedures to impute an ICC. For example, Puzio et al.^19^ imputed a value of 0.2 for an ICC approximation based on ICC values that Hedges and Hedberg^13^ reported, where the mean (across grade level) ICC for reading outcomes was 0.224. In another example, Graham et al.^20^ took the median of the ICC values across all educational outcomes reported in Hedges and Hedberg.^13^ The What Works Clearinghouse Procedures Handbook v. 5.0^11^ recommends imputing an ICC value of 0.20 for achievement outcomes and 0.10 for behavioral outcomes. However, those values may be inaccurate and may not be relevant to all outcome subtypes and study designs more generally. And as noted earlier, inaccurate ICCs can negatively impact results from a priori power and meta-analytic inferences.

#### 2.3.2 Confidence interval estimates of the ICC

Instead of only imputing a value from a previous study of similar design, other researchers have suggested adjusting for potential uncertainty in the ICC parameter by constructing confidence interval estimates and using the upper limit as the imputed value.^21^ Using the variance formulas discussed in detail later, confidence intervals for the ICC value can be constructed. To avoid under-powering a study, applied researchers could use the upper limit of a 95% confidence interval estimate of ρ as a conservative ICC estimate to substitute in a power analysis. However, use of the upper limit value can project a larger than necessary sample size^21^ and result in less accurate adjustments when meta-analyzing effect sizes from clustered data.

#### 2.3.3 Utilizing Bayesian methods to estimate ICC values

Another proposed method of obtaining ICC estimates utilizes a Bayesian framework, which is not often implemented by applied researchers in social science research. Instead of hypothesizing a single true ICC value, a plausible prior distribution can be specified to derive a posterior distribution of ICC values. To capture reasonable ICC estimates for analyses, Spiegelhalter^22^ discussed the use of eight different priors that could be placed on the multilevel model’s variance components and the ICC estimate (Equation (2.2)). Spiegelhalter^22^ evaluated the priors through a Markov chain Monte Carlo (MCMC) demonstration and found that the inverse gamma priors, the log-uniform prior, and the uniform shrinkage prior all led to substantially smaller estimates of the ICC, which would result in inflated statistical power in the prospective designs. Findings from this study support using the uniform prior for the ICC, or the uniform shrinkage prior, among the uninformative prior options when estimating an ICC. The work of Spiegelhalter^22^ relies on the availability of the raw data in order to specify the likelihood function. However, when conducting power analyses or meta-analyses, previous estimates of ICCs are rarely accompanied by the raw data, and researchers must rely on summary statistics instead.

To develop a method that relies on summary statistics instead of raw data when estimating an ICC, Turner et al.^23^ compared possible forms for the likelihood function of ICC estimates using methods described in Donner and Wells^21^ and Ukoumunne.^49^ The proposed likelihood distribution functions do not rely on raw data and only require values for the ICC estimate, the total number of observations, and the total number of clusters. Turner et al.^23^ conducted an empirical MCMC demonstration to construct posterior distributions for the true ICC value using the various forms of the likelihood when paired with a Uniform(0, 1) prior. Then they transformed the resulting posterior distribution into a distribution of power values. As noted earlier and discussed in greater detail later, the distributional form of an ICC is unknown, and the methods of Turner et al.^23^ rely heavily on the assumptions made about the likelihood. Furthermore, her demonstration only used one or six ICC estimates, so the conclusions were entirely dependent on the prior and likelihood choices. Thus, while there are a number of ad hoc suggestions for imputing an ICC estimate, there are challenges identified with each. In the following sections, we describe how a more principled synthesis of prior ICC estimates could be used to impute a reasonable value.

### 2.4 Characteristics of the sampling distribution of ICCs

Pooling ICC estimates requires specifying a sampling variance for the ICC. Despite the sampling distribution of the ICC being unknown due to it being a ratio of variances, several methods have been proposed to construct confidence intervals for the ICC to capture uncertainty in the parameter. In the scenario where there are equal cluster sizes and large samples, there are closed-form approximations for constructing confidence intervals of ICC estimates.^1^ ^,^ ^50^ For designs with unequal cluster sizes, closed-form approximations for confidence intervals of ICCs have been proposed assuming either an F-statistic^51^ or a large-sample approximation to the variance of an ICC estimate. Ukoumunne^49^ and Donner and Wells^21^ detail several methods for constructing confidence intervals for ρ where the sampling distribution of ICC estimates and transformed ICC estimates are assumed to follow a normal distribution.^1^ ^,^ ^2^ ^,^ ^26^ ^,^ ^27^ ^,^ ^52^

There have been studies that explored the empirical ICC distributions based on real data using observed variability and simulated data using assumed models and parameters. The shape of the empirical distribution of ICC estimates depends on the magnitude of the true ICC parameter and data context. For example, Hedberg and Hedges^15^ assessed the empirical distribution of 3,555 within-district ICC estimates they collected and found it to be non-normal and highly positively skewed with a median ICC value of 0.056 . But, as they noted, the empirical distribution of ICC estimates in their context accounts for both the sampling variance of a school-level ICC estimate and between-district variance (comparable to a between-study variance in a meta-analytic context), which could impact the shape of the distribution. Furthermore, when ICC estimates are near zero, the estimates would pile up near the lower bound of zero.

Simulation studies that do not assume a distribution of population ICC estimates, but instead assume only a single underlying ICC parameter value, can evaluate the shape of the ICC estimates’ sampling distribution by using an empirical distribution of simulated ICC estimates under specified models or other parameters. Liljequist et al.^53^ conducted a Monte Carlo simulation to evaluate ICC distributions in the context of ICC as a measure of reliability. They assumed that the level-1 and level-2 residual terms were normally distributed and calculated an ICC value using an ANOVA model. When the true ICC value was 0.8, the empirical distribution was highly left-skewed, and when the true ICC value was 0.5 or 0.64, the resulting empirical distributions of ICC estimates were more symmetric but still skewed to the left. The authors also evaluated the impact of increasing the number of cluster units (participants in this context) and found that the empirical distribution becomes narrower as the number of clusters increases. They also examined the magnitude of within-cluster units (measurements in this context). The values of the number of within-cluster units they evaluated ranged from 2 to 10 measurements per subject, which is small in comparison to the values of the number of within-cluster units in CRT contexts. They found that increasing the number of within-cluster units also decreases the width of the empirical distribution.

Assumptions about the ICC sampling distribution following a normal distribution are likely often violated in practice (particularly for smaller samples) and negate the validity of using confidence interval estimates of the ICC, which have traditionally assumed a normal distribution for ICC estimates.

Regarding the sampling variance, $v_{\hat{\rho }}$ , of an ICC estimate, researchers have derived different formulas for large samples. Smith^2^ proposed a large sample approximation to the variance of the ICC estimate by using the weighted mean cluster size ( $n_{0}$ ):

(2.3) $$\begin{array}{r} n_{0}=\frac{1}{j-1}\left[N-\sum_{i=1}^{j}\frac{n_{i}^{2}}{N}\right], \end{array}$$

where *j* is the number of clusters, *N* is the total sample size, and $n_{i}$ is the number of units in the *i*th cluster. The formula derived by Smith^2^ for the variance of an ICC estimate is as follows:

(2.4) $$\begin{array}{r} \begin{array}{rl} v_{\hat{\rho }} & =\frac{2(1-\hat{\rho }{)}^{2}}{n_{0}^{2}}\{\frac{[1+\hat{\rho }(n_{0}-1){]}^{2}}{\left(N-j\right)}\\ & \;+\frac{(j-1)(1-\hat{\rho })[1+\hat{\rho }(2n_{0}-1)]+{\hat{\rho }}^{2}\left[\sum n_{i}^{2}-2N^{-1}\sum n_{i}^{3}+N^{-2}{\left(\sum n_{i}^{2}\right)}^{2}\right]}{(j-1{)}^{2}}\}, \end{array} \end{array}$$

where $\hat{\rho }$ is an estimate of ρ .

The formula for $v_{\hat{\rho }}$ proposed by Swiger et al.^26^ is a variation of Smith’s approach, Equation (2.4), with a simpler formula for the variance of the ICC estimate:

(2.5) $$\begin{array}{r} v_{\hat{\rho }}=\frac{2(N-1)(1-{\hat{\rho }}^{2})[1+(n_{0}-1)\hat{\rho }{]}^{2}}{n_{0}^{2}(N-j)(j-1)}. \end{array}$$

Using the delta method, Hedges et al.^27^ derived the large sample variance of an ICC estimate for a two-level model in designs with equal cluster sizes as follows:

(2.6) $$\begin{array}{r} v_{\hat{\rho }}=\frac{(1-\hat{\rho }{)}^{2}v_{2}}{(\sigma_{e}^{2}+\sigma_{u}^{2}{)}^{2}}, \end{array}$$

where $v_{2}$ is the variance of $\sigma_{u}^{2}$ . The variance of the level-2 variance is commonly not reported in primary research studies, so applied meta-analysts will not be able to implement it, unlike other formulas that do not require it.

Hedges et al.^27^ stated that in balanced designs with equal cluster sizes, Equation (2.6) is equivalent to the formulation derived by Fisher^1^ for large samples. The formula derived by Fisher^1^ is as follows:

(2.7) $$\begin{array}{r} v_{\hat{\rho }}=\frac{2(1-\hat{\rho }{)}^{2}[1+(n-1)\hat{\rho }{]}^{2}}{n(n-1)(j-1)}, \end{array}$$

where *n* is the average cluster size.

For designs with unequal cluster sizes, Donner and Koval^52^ derived a formula for the large sample variance of ρ estimates as follows:

(2.8) $$\begin{array}{r} v_{\hat{\rho }}=\frac{2N(1-\hat{\rho }{)}^{2}}{N\sum_{i=1}^{j}n_{i}(n_{i}-1)\hat{V_{i}}{\hat{W_{i}}}^{-2}-{\hat{\rho }}^{2}[\sum_{i=1}^{j}n_{i}(n_{i}-1){\hat{W_{i}}}^{-1}{]}^{2}}, \end{array}$$

where $\hat{W_{i}}=1+(n_{i}-1)\hat{\rho }$ and $\hat{V_{i}}=1+(n_{i}-1){\hat{\rho }}^{2}$ . With equal cluster sizes, when $n_{1}=\cdots =n_{j}=n$ , Equation (2.8) reduces to Fisher’s formulation, Equation (2.7).

Finally, there is also Fisher’s method using a large sample approximation to the SE of a normalizing transformation of ρ .^29^ First, the ICC estimates are assumed to be normally transformed using the following:

(2.9) $$\begin{array}{r} Z_{F}=\frac{1}{2}\ln\frac{1+(n_{0}-1)\hat{\rho }}{1-\hat{\rho }}, \end{array}$$

and the corresponding variance formula is

(2.10) $$\begin{array}{r} v_{\hat{\rho }}=0.5\{(j-1{)}^{-1}+(N-j{)}^{-1}\}. \end{array}$$

In summary, Fisher^1^ derived a formula for $v_{\hat{\rho }}$ that requires the ICC estimate, the average cluster size, and the number of clusters. Swiger et al.^26^ presented a formula that is the same as Fisher’s formula except scaled by a function of the total number of individuals in the sample. Hedges et al.^27^ derived a formula requiring knowledge of the variance of the level-2 random effects variance for a primary study, which is often not reported in primary studies. Donner and Koval^52^ and Smith^2^ derived formulas for unbalanced cluster sizes; the Donner and Koval^52^ formula is equivalent to Fisher’s formula when cluster sizes are equal. Fisher^29^ presented a formula that uses a normalizing transformation of the ICC estimate and requires only the weighted mean cluster size and the ICC estimate. The aim of this study is to compare how well these variance formulas work when quantitatively synthesizing ICC estimates.

Many simulation studies have compared confidence interval methods for single (not pooled) ICC estimates, which implicitly evaluate the performance of the different sampling variance formulas. Among the formulas we presented above, Donner and Wells^21^ examined those proposed by Swiger et al.,^26^ Fisher,^29^ and Smith^2^ by assessing confidence interval coverage probabilities for ρ when the number of lower-level units within clusters varied across clusters (e.g., unbalanced design). They found that the Swiger formula^26^ had excellent coverage for ρ values less than 0.3 and adequate coverage up to when ρ=0.7 . Additionally, the Smith formula^2^ performed well across all values of ρ , and they recommended its use in practice. In contrast, the Fisher normalizing transformation formula (Fisher TF)^29^ consistently performed conservatively relative to the other variance formulas they evaluated.

In the context of CRTs for values of ρ≤0.3 , Ukoumunne^49^ compared the performance of the Smith formula, Swiger formula, Fisher large samples formula (Equation (2.7)),^1^ and Fisher TF formula in an unbalanced design. They found that although the Smith estimator provided better coverage than the other formulas based on the untransformed ICC, the Fisher TF formula had substantially higher coverage across all large-sample approximations they evaluated. Coverage for the Fisher TF formula also improved as the number of clusters increased. In contrast, the variance formulas for the untransformed ICC performed particularly poorly in the condition with a small number of clusters ( j=10 ) and small average cluster size ( $n_{0}=10$ ), where coverage proportions for all three methods fell below 90%. Overall, Ukoumunne^49^ also found that the precision of all confidence interval methods improved as the average cluster size and the number of clusters increased, but worsened as the magnitude of the ICC parameter increased.

Demetrashvili et al.^54^ also evaluated confidence intervals for the ICC from a one-way random effects model, where they proposed new approaches (one assuming an F-distribution and using the Satterthwaite approximation, and another assuming a Beta distribution). They compared the method proposed by Searle,^51^ the Fisher TF formula, and the Smith formula across a large range of ICC parameter values (0.1–0.9). In conditions where the number of level-1 units was smaller than the number of level-2 units, the authors found that the Smith formula had poor confidence interval coverage rates. They also found that the Fisher TF formula resulted in conservative coverage probabilities across all values of ρ that they evaluated.

Across all of these methods, it is unclear which formulation, when used for meta-analytic pooling, would result in an accurate pooled ICC estimate. Furthermore, it is unknown whether the distributional assumptions made by these methods are accurate, since the form of the sampling distribution of the ICC has not been derived. Methods for pooling ICC estimates that are agnostic to distributional form, therefore, may provide an ideal alternative because they do not require strong assumptions about the distributional shape of the statistic being pooled.

### 2.5 Meta-analytic methods to pool ICC estimates

In the next section, we review the most commonly used meta-analytic methods of relevance to this study. Ultimately, the hope is that an applied researcher could use these methods to pool ICC estimates from prior studies to be imputed for use in a secondary analysis (such as a power analysis of CRTs or meta-analysis of effects from clustered data), and that the resulting pooled ICC reflects the relevant population and research context.

One of the fundamental applications of meta-analysis entails pooling effect sizes from primary studies to obtain an overall effect size for the set of studies included in the analysis. The type of effect size varies depending on the type of data and the research questions of the primary studies, and most typically includes SMDs, log odds ratios, and correlations.^55^ Typically, an effect size is assumed to follow a normal distribution (otherwise, it is transformed to follow an approximate normal distribution) with a known sampling SE.^56^ The standardized effect size for study *i* is generically represented as $T_{i}$ . For this study, the ICC estimate is the “effect size” that is being pooled using meta-analytic techniques.

In meta-analytic pooling, assuming that there are *k* studies included in a meta-analysis, that each study reports one independent effect size (simplest scenario), $T_{i}$ , for studies i=1,…,k . The effect sizes are pooled using the following:

(2.11) $$\begin{array}{r} \hat{\mu }=\frac{\sum_{i=1}^{k}w_{i}T_{i}}{\sum_{i=1}^{k}w_{i}}, \end{array}$$

where $w_{i}$ are model-dependent weights specified by the analyst. The most typical weight applied is the inverse of the effect size’s variance estimate.

#### 2.5.1 Using REML estimation to estimate the pooled ICC

While there are many estimation methods for random effects meta-analytic models, REML is the default of the meta-analytic R software package *metafor* ^57^ and is commonly used in most meta-analysis packages. Unlike other maximum likelihood estimators that negatively bias the effect size estimates’ variance component, REML corrects for this and has been adapted for meta-analytic random effects pooling.^58^ ^,^ ^59^

#### 2.5.2 Using robust variance estimation to estimate the pooled ICC

RVE^34^ is an alternative meta-analysis regression model estimation approach. It is typically employed to account for the dependence among multiple effect sizes within studies when estimating SEs. More generally, RVE uses the weighted least squares (WLS) framework, which incorporates a set of weighting matrices in estimation. It was adapted by Tipton and Pustejovsky^60^ for meta-analyses involving only a small number of studies, using the Satterthwaite degrees-of-freedom correction. When there is no dependence among the effect sizes, as is the case with a random effects model with independent effect sizes, RVE can also be used to obtain robust estimates of the variance of the pooled effect because RVE is robust to the distributional shape of the effect size being pooled.^34^ RVE estimates parameters using method-of-moment estimators proposed by Hedges et al.^34^

RVE does not require knowledge of the exact covariance structure between pairs of effect size estimates because the variances of the meta-regression coefficients are estimated with sandwich estimators using the cross-product of observed residuals to estimate the covariance between pairs of effect sizes rather than the known covariances.^34^ ^,^ ^60^ RVE is used with a variety of data structures, including those that are nested or correlated.

Even though any set of weights can be used, the inverse variance weights are typically specified because if the working model is correct, using inverse variance weights will result in a fully efficient estimator.^34^ There are different formulations of the inverse variance weights depending on the assumptions made about the working model.^34^ Because the true covariance structure between effect sizes is typically unknown, exact inverse variance weights cannot be calculated. The approximate inverse variance weights improve efficiency compared to any other type of weights.^34^

Since we evaluate only one ICC per study in this study, the working model will also be a random-effects meta-regression model, and the weight will be specified as, where ${\hat{v}}_{\hat{\rho }i}$ is the sample variance estimate for $\hat{\rho }$ from study i and ${\hat{\tau }}_{\rho }^{2}$ is the between-study variance estimate for the ICC $W_{i}=\frac{1}{\left({\hat{v}}_{\hat{\rho }i}+{\hat{\tau }}_{\rho }^{2}\right)}$ . However, instead of using REML for estimation, Hedges et al.^34^ used method of moments estimation to estimate the between-study variance $\tau_{\rho }^{2}$ and the weights in the RVE technique. So, essentially, when the working model is a random effects model with independent effect sizes (as we use in the current study), the comparison between RVE and REML is really a comparison between REML and the method of moments estimation of the between-study variance of the ICC, $\tau_{\rho }^{2}$ .

RVE has two advantages over typical meta-analytic pooling that uses estimation methods like REML. First, the RVE estimate of the variance of the weighted mean effect size is an unbiased estimate regardless of the choice of weights.^34^ The lack of reliance on the choice of weights means a lack of dependence on the effect size estimate variance specification because the weights are typically a function of those variances. Second, RVE performs well without requiring strict distributional assumptions for analyses of effect size estimates. Because the aim of this study is to pool ICC estimates (as the “effect sizes” being meta-analytically pooled), these advantages are useful because the distributional form and the variance of the sampling distribution of ICC estimates are unknown. For optimal performance, other models or methods of pooling rely on distributional assumptions or weight specification for accurate estimation of parameters.

#### 2.5.3 Applied meta-analyses of ICC estimates

Meta-analytically pooling ICC estimates is not a novel notion. Applied researchers have used these methods to find representative ICC values for specific research designs, outcome types, and populations. The methods used for meta-analyzing ICC estimates in practice vary widely.

For example, as mentioned earlier, Hedberg and Hedges^15^ meta-analyzed ICC values collected from districts estimated from raw data. The two-level ICC values in the dataset were estimated for each school within each district using REML. Then, the authors used the random effects model to pool the within-district ICCs estimates across districts (the random effects) using a method of moments estimator derived by Hedges and Vevea^61^ to account for the sampling error and the variance in the district ICC values around the population average ICC. For the variance of the ICC, they used the large sample variance derived by Fisher^1^ (see Equation (2.7)).

In another applied example of a meta-analysis of ICC estimates, Kivlighan et al.^30^ meta-analyzed ICCs from CRTs of group therapy. The meta-analysis summarized 169 ICC estimates from 37 studies using RVE. The researchers used RVE to handle the within-study dependency in ICCs resulting from multiple ICC values being reported per study. For the inverse variance weights, the authors estimated the variance of the ICC, $v_{\hat{\rho }}$ , using the formula proposed by Hedges et al.,^27^ which requires the variance of the cluster-level variance. However, there are several limitations in the methods used by Kivlighan et al.,^30^ which should be considered when interpreting their results. First, the authors did not provide the exact form of the variance they used in the meta-analysis; they only provided the weights, so we could not verify how they were able to calculate the variances of the ICC estimates. They did not detail any assumptions they made about the variance of the cluster-level variances used when calculating the ICC estimate variances. Furthermore, they pooled level-2 ICC estimates and level-3 ICC estimates together. The range of ICC estimates at different nesting levels has been found to vary,^13^ ^,^ ^14^ so pooling ICC estimates from different levels of an MLM is not appropriate.

In the context of an ICC as a reliability measure, Noble et al.^31^ meta-analyzed 25 independent ICC estimates as a measure of test–retest reliability of fMRI-based functional connectivity using a multilevel model (to account for studies nested with datasets) with REML estimation using a modified version of the Fisher formula (Equation (2.7)) presented in Shoukri et al.^43^ However, they also mention that they used this sample variance formula for other types of ICCs included in their meta-analysis, beyond the one-way random-effects ICC.

Goerdten et al.^33^ meta-analyzed ICC estimates as a measure of reproducibility over time for a biomarker involved in metabolism (metabolites). They also used a multilevel random-effects model to account for a study reporting multiple ICC estimates (with effects nested within authors). They did not specify the estimation method, but they conducted their analyses in metafor, which uses REML as a default. For the sampling variance for the ICC estimate, they also used the modified version of the Fisher formula presented in Shoukri et al.^43^

Nicolaï et al.^32^ also meta-analyzed 29 ICC estimates across eight studies to assess the most reliable treadmill protocol for patients with peripheral arterial disease. The authors used a fixed effects meta-regression model using the Fisher TF formula for the ICC sampling error variance and accounted for the covariance of ICC estimates that were dependent using the equation for covariance given by Donner and Zou.^62^

Regardless, many applied meta-analyses of ICC estimates are using a variety of meta-analytic modeling methods and different variance formulas in the formulation of the weights. More insight is needed on how best to pool ICCs, including which formulations, assumptions, and methods work best.

#### 2.5.4 Methodological research on meta-analyses of ICC estimates

Across two simulation studies, Ahn et al.^63^ evaluated the synthesis of SMD effect sizes when the primary studies were a mix of research designs, including 2-level clustered and individual randomized design studies. To compute the ICC needed for correcting effect sizes for clustering, the authors pooled the log-transformed cluster and total standard deviations (separately).^4^ The first simulation study aimed to see how well the true ICC was captured by pooling the sampled studies’ standard deviation components. The authors fixed the number of clusters and individuals per cluster to 30 (not evaluating other cluster or per-cluster sizes) and varied the population ICC value, mean differences between treatment and control, and the number of cluster-level and individual-level studies in the sample. Across these conditions, they found that the estimated ICC was unbiased and fairly precise using relative bias and mean squared error as performance criteria.

The second simulation that the authors conducted evaluated the use of the estimated ICC in a meta-analysis. They generated a meta-analytic dataset and varied the number of studies with and without clustering, population ICC value, population mean difference, and total number of studies in the meta-analysis. They found that the overall effect size estimates that resulted from using a default ICC of 0.2 or from not correcting for clustering at all were both substantially biased.

In this study, the authors did not detail the estimation procedure they used for obtaining the cluster-level and total variance components used to calculate each study’s ICC. In addition, to pool standard deviations across studies, the scale of the outcome has to be assumed constant across all studies in the meta-analysis. This greatly limits the use of the authors’ method for synthesizing ICCs using the reported cluster and total variances. However, the substantial bias found when a generic rather than a more accurate ICC value is used for correcting effect sizes for clustering is critical and relevant to this study. These findings highlight the importance of determining the optimal procedure for pooling ICCs to obtain an accurate ICC estimate.

In summary, the methods that have been proposed to estimate an ICC from external sources (primary studies) are less than ideal. Primarily, the methods each include one or more of the four limitations: (1) reliance on the availability of raw data, (2) requirement of strong assumptions of distributional form for the sampling distribution of ICC estimates, (3) potentially biased/imprecise ICC estimates, and (4) overly conservative ICC estimates. One potential method for addressing these shortcomings is a pooling technique typically applied to meta-analysis of effect sizes.

Limited work has been done to evaluate meta-analytic estimation methods used when pooling ICCs. In particular, best practice for specifying weights requires additional attention and guidance. Meta-analytic weights are typically a function of the effect size’s sampling error variance. Because multiple formulations for the ICC’s variance have been suggested, research on the optimal formulation for this variance is also needed.

The current study aims to address the following research question: How well does pooling of ICC estimates capture the population ICC value a) under a variety of conditions when assuming a random effects model paired with b) method of moments versus REML estimation and when c) both estimation procedures are used with different ICC estimate variance formulas in the inverse variance weights?

### 2.6 An application of meta-analyzing ICC estimates

To assess the performance of the various formulas for the sampling variance of an ICC and the estimation methods for the random effects model, we meta-analyzed a public dataset published by the Massachusetts Comprehensive Assessment System (MCAS). The dataset includes raw English Language Arts scores and mathematics test scores for each student. From raw student data, we estimated a two-level ICC estimate at the school level within each school district with five or more schools using an MLM with REML estimation. Using raw data for this demonstration enables the computation of all the sample variance formulas. However, when gathering ICC estimates from primary studies, the Smith,^2^ Hedges,^27^ and Donner^52^ formulas will be difficult to implement due to what is primarily reported in primary studies.

**Figure 1:**
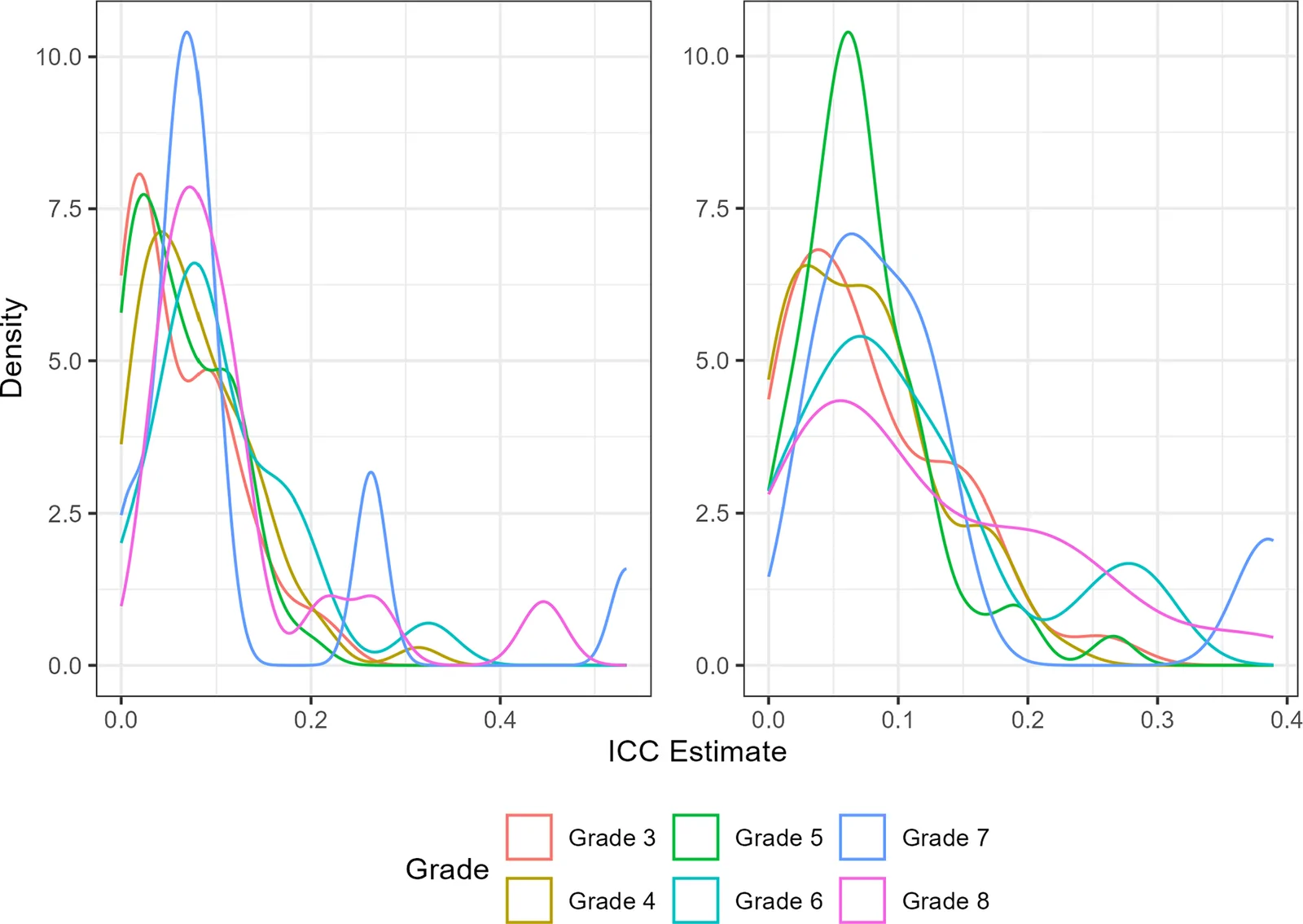
Empirical distributions of ICC estimates by grade level for English Language Arts scores (left) and mathematics scores (right). Figure 1 Long description.

Figure 1 depicts the density distributions of the ICC estimates for each grade. The graph on the left shows the density plots of the ICC estimates when English Language Arts scores were the outcome, and the graph on the right shows the density distributions when mathematics scores were the outcome. As was the case for Hedberg and Hedges,^15^ these distributions have sources of variation from both sampling error and between-district variability. Due to these sources of variation, these empirical distributions may be a good representation of the type of data one might collect for a meta-analysis of ICC estimates from different primary studies, since between-district variability is comparable to between-study variability. The resulting empirical distributions were positively skewed.

For each grade (grades 3–8), we meta-analytically pooled the ICC estimates using RVE or REML in combination with the six different inverse variance weights formulations. For this example, we evaluate only the overall pooled ICC estimates to be ultimately used in secondary analyses.

**Figure 2:**
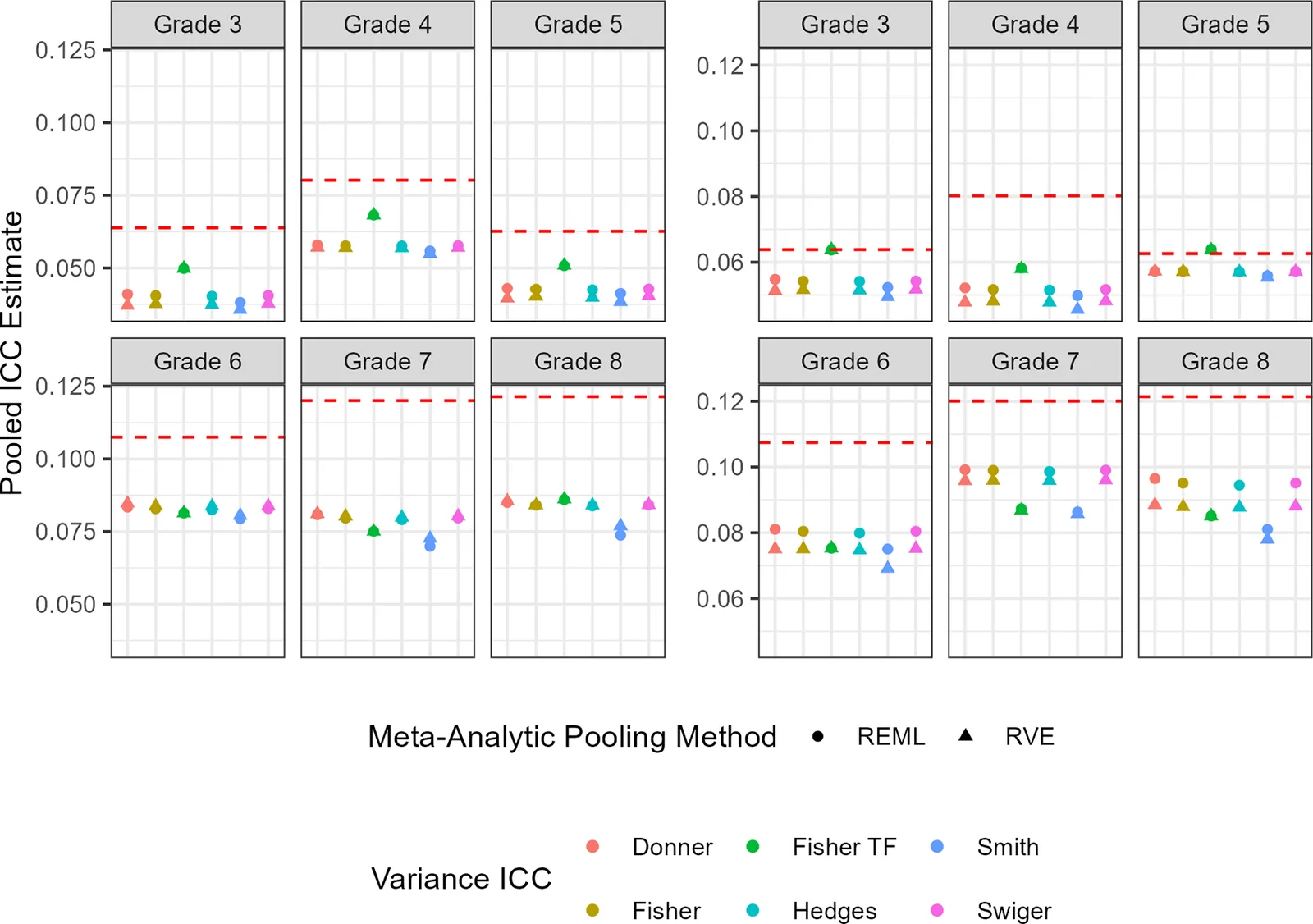
Pooled ICC estimates by grade level using either REML or RVE estimation in meta-analytic pooling in combinations with six different inverse variance weights for English Language Arts scores (left) and mathematics scores (right). *Note*: Dotted redline represents the arithmetic mean of the ICC estimates. Figure 2 Long description.

Figure 2 displays the graphs (left has the results for the English Language Arts scores and right has the results for the mathematics scores) of the overall pooled ICC estimates for each grade using either RVE or REML in combination with the six variance formula as weights. The dotted red line represents the arithmetic mean of the ICC estimate. Results demonstrate that REML and RVE differ slightly in some of the grades when the distributions of ICC estimates are more spread out, as is the case with the mathematics scores for grades 3, 4, 6, 7, and 8. There were no differences between REML and RVE when the distributions of ICC estimates are more peaked (grade 5 for mathematics scores and grades 4, 6, 7, and 8 for the English Language Arts scores), regardless of the number of ICC estimates pooled.

Among the different formulas for the ICC estimate of the sample variance, only the formula that uses the normalizing transformation of ICC^29^ and the Smith formula^2^ yield notably different pooled ICC estimates when used as inverse variance weights. Furthermore, when using the Fisher TF formula in the inverse variance weights, RVE and REML estimation both result in the same pooled ICC estimate. In most cases, when the results for RVE and REML differ, pooled ICC estimates from REML are closer to the arithmetic mean of the ICCs.

## 3 Methods

To study the performance of various meta-analytic methods for pooling ICC estimates, we conducted a Monte Carlo simulation study that replicates realistic conditions under which such methods may be employed. We evaluated whether RVE^34^ or REML^35^ with random-effects pooling and different weight specifications resulted in unbiased ICC parameter estimates by creating meta-analytic datasets of ICCs estimated from raw data. We first generated raw data with a two-level nested structure for each primary study, while adding in between-study heterogeneity. Then, for each primary study in the simulated meta-analysis, we estimated the ICC using REML estimation for the variance components and then used the estimates of those variances to compute the study’s ICC estimate. We also calculated the variance of the ICC estimate using several different formulas (see Equations (2.4)–(2.10)) to use as weights in the meta-analytic models. Finally, for each combination of conditions, we then built meta-analytic datasets of ICC estimates from each primary study to meta-analyze the ICCs and compare to the true ICC value used to generate the raw data.

### 3.1 Data generation method

After generating between-study heterogeneity in the true ICC estimates, as detailed below in Section 3.2, we generated raw data for each primary study using a two-level unconditional random effects model (see Equation (2.1)). We sampled the cluster level residuals from $∼N(0,\;\sigma_{u}^{2})$ and the individual unit level residuals from $∼N(0,\;\sigma_{e}^{2})$ . The values of $\sigma_{u}^{2}$ and $\sigma_{e}^{2}$ were manipulated as part of the simulation study. The value of the true intercept value, $\gamma_{00}$ , was set to equal zero (see Equation (2.1)).

We varied the number of clusters, *j*, and average number of units per cluster, ${\overset{―}{n}}_{j}$ , because sample size has been found to affect the recovery of MLM variance components.^64^ ^,^ ^65^ We induced imbalance in the cluster sizes in order to evaluate the variance of the ICC estimate in scenarios with unbalanced cluster sizes.^52^

Accurate estimation of the variance components from the original primary study’s raw data that are used to calculate the ICC is vital to reduce bias when pooling the ICC estimates. We used REML to estimate the level-1 and level-2 variance components (see Equation (2.2)) and their associated variances because REML is often a default of many software packages. Then, we calculated the ICC value from the variance components and calculated the variance for the ICC using each of the formulas presented above. This process was repeated until the meta-analytic dataset was fully populated with an ICC estimate for each primary study generated.

### 3.2 Experimental design

In order to evaluate a large range of datasets with clustered data, we compared meta-regression model estimation methods paired with different ICC variance formulas for pooling ICCs for datasets created through manipulation of the number of clusters, number of individuals per cluster, the degree of balance between clusters, the true ICC values, the magnitude of between-study heterogeneity, and the number of ICC estimates to pool.

#### 3.2.1 Number of clusters in raw data, *j*

The recovery of MLM parameter estimates, their SEs, and variance components is affected more by the number of clusters than by the number of individuals per cluster.^64^ ^,^ ^65^ For each primary study in a meta-analytic dataset, we sampled the number of clusters from a uniform distribution in order for it to vary across primary studies within a meta-analytic dataset. We chose a uniform distribution to be agnostic about the distribution of the number of clusters in multilevel data. We investigated two ranges of values for the number of clusters per primary study in a meta-analytic dataset: 30–50 or 50–100, representing a range of values common in applied cross-sectional multilevel research.^66^

#### Number of individuals per cluster, $n_{j}$

3.2.2

We also varied the number of individuals per cluster across primary studies in a meta-analytic dataset by drawing from a uniform distribution because the optimal distribution to assume for cluster sizes is unknown. We used the following values for the bounds of the uniform distribution of average cluster sizes, ${\overset{―}{n}}_{j}$ , 10–30 or 30–50, reflecting the typical range of cluster sizes found in other multilevel simulation studies.^66^ ^,^ ^67^

Because two of the variance formulas account for unequal cluster sizes (see Equation (2.8)^28^ and see Equation (2.4)^2^), and it is reasonable to assume unequal cluster sizes, we decided that the primary studies’ datasets should reflect this reality of applied datasets. To induce imbalance in the cluster sizes, we sampled the cluster sizes from a uniform distribution where the minimum and maximum values were a fixed proportion below and above the average sample size: $n_{j}∼Unif[{\overset{―}{n}}_{j}(1-\zeta ),\;{\overset{―}{n}}_{j}(1+\zeta )],$ where ${\overset{―}{n}}_{j}$ is the condition’s average per cluster sample size for cluster *j* and ζ is the proportion of ${\overset{―}{n}}_{j}$ that is added to and subtracted from the average cluster size to obtain the bounds of the uniform distribution. By doing this, the size of each cluster was different, with the value falling within the range of $\left[{\overset{―}{n}}_{j}(1-\zeta ),\;{\overset{―}{n}}_{j}(1+\zeta )\right]$ . We decided to set the boundaries for the uniform distribution to be ζ=0.5 and ζ=0.1 of the ${\overset{―}{n}}_{j}$ to vary the degree of imbalance in the primary dataset.

#### True ICC values, ρ

3.2.3

For the true population values of the intraclass correlation, we used 0.05, 0.10, 0.15, 0.25, 0.50, and 0.90. We chose ρ=0.05,0.15, and 0.25 based on a review of ICCs found in educational research. Hedges and Hedberg^13^ compiled databases of ICC values with values ranging from 0.045 to 0.271 in educational research settings. Simulation studies that varied ICC as a condition referenced Hedges and Hedberg’s study^13^ using ICC values within that range.^67^ ^–^ ^69^ An ICC value of 0.05 would be considered a relatively smaller value, while an ICC value of 0.25 would be considered a relatively large ICC for educational research involving clustered individuals’ data. We also added ρ=0.1 since The What Works Clearinghouse Procedures Handbook v. 5.0^11^ suggests imputing this value as a conservative ICC value for behavioral outcomes. The ICC values of 0.5 and 0.9 would be more meaningful when ICC is used as a reliability measure with typically higher values than seen for ICCs for clustered individuals’ data.

*Variance components of the true ICC at Level-1*, $\sigma_{e}^{2}$ , *and Level-2*, $\sigma_{u}^{2}$ : We manipulated the values of $\sigma_{u}^{2}$ and $\sigma_{e}^{2}$ to have a total variance of 100. Solving for $\sigma_{u}^{2}$ in Equation (2.2), we determined the value of the level-2 variance for each condition by multiplying the total variance by the ICC value as specified by the condition. Then we found the level-1 variance by subtracting the level-2 variance from the total variance value of 100. Table 1 displays the combinations of level-1 and level-2 variances for respective ICC values of 0.05, 0.10, 0.15, 0.25, 0.50, and 0.90 and a total variance of 100. We used the nlme package^70^ to estimate the variance components.

**Table 1:** Condition-specific variance values Table 1 Long description.

Total variance	True intraclass correlation	Level-1 variance	Level-2 variance
$\sigma_{u}^{2}+\sigma_{e}^{2}=100$	ρ=0.05	$\sigma_{e}^{2}=95$	$\sigma_{u}^{2}=5$
	ρ=0.10	$\sigma_{e}^{2}=90$	$\sigma_{u}^{2}=10$
	ρ=0.15	$\sigma_{e}^{2}=85$	$\sigma_{u}^{2}=15$
	ρ=0.25	$\sigma_{e}^{2}=75$	$\sigma_{u}^{2}=25$
	ρ=0.50	$\sigma_{e}^{2}=50$	$\sigma_{u}^{2}=50$
	ρ=0.90	$\sigma_{e}^{2}=10$	$\sigma_{u}^{2}=90$

#### Between-study heterogeneity, $\tau_{\rho }$

3.2.4

To introduce between-study heterogeneity ( $\tau_{\rho }$ ) into the ρ estimates, we used a uniform distribution. We choose a uniform distribution for the population distribution for the ICC parameter, because, in this study, we chose to be agnostic about the shape of the distribution of the population of ICCs. We are primarily interested in the ICC’s sampling distribution and, in particular, in the sampling variance of the ICC that works best for the meta-analytic pooling ICC estimates. The boundaries of the distribution were $\rho \pm 2\tau_{\rho }$ . For the values of $\tau_{\rho }$ , we analyzed a dataset that includes raw English Language Arts and mathematics test scores for students participating in the MCAS. For each district with five or more schools, we estimated school-level ICCs using a two-level model. Then, we pooled the ICC estimates by grade level using REML and RVE estimation with the various variance formulas as weights (Equations 2.4–2.10). We found values of $\tau_{\rho }$ estimates between 0.026 and 0.058 and a median of 0.037. From these values, we obtained the scale of $\tau_{\rho }$ for the ICCs. Since our smallest true ICC has a value of 0.05, and to avoid generating negative values for any ICC, we chose smaller values of $\tau_{\rho }$ of 0.01 and 0.02.

#### 3.2.5 Number of primary studies, *k*

For the number of primary studies to pool, we generated meta-analytic database sizes of 20, 50, and 100 studies. For the upper range of the number of ICC estimates to pool, we chose 150 since that is around the number collected by the applied meta-analysis of ICC estimates we found^30^ and represents a relatively large applied meta-analysis. We generated one ICC value per study as a starting point for this line of research. Future research can explore scenarios in which there might be multiple ICC estimates per study.

#### 3.2.6 Experimental factors

Table 2 lists the values for each condition examined in the study. The following factors are included in the study: 2 values for the number of clusters × 2 values for the average number of individuals per cluster × 2 values for the degree of imbalance × 6 values for $\sigma_{e}^{2}$ and $\sigma_{u}^{2}$ combinations × 3 values for number of pooled ICC estimates × 2 values for the between study heterogeneity, totaling 288 conditions. For the simulation, we ran enough replications to ensure that there were 1,000 replications for which each estimation procedure converged.

**Table 2:** Data generating conditions Table 2 Long description.

Conditions	Values
Number of clusters in raw dataset (*j*)	U [30, 50], U [50, 100]
Average number of units per cluster ( ${\overset{―}{n}}_{j}$ )	U [10, 30], U [30, 50]
Degree of unbalance, ζ	0.1, 0.5
Level-1/Level-2 variance ( $\sigma_{e}^{2}/\sigma_{u}^{2}$ )	95/5; 90/10; 85/15; 75/25; 50/50; 10/90
Number of primary studies (*k*)	20, 50, 100
Between study heterogeneity ( $\tau_{\rho }$ )	0.01, 0.02

*Note*: This study assumes one ICC value per primary study.

In addition, for each simulated dataset, we pooled the ICC estimate using a random effects meta-analytic model^71^ with two different estimation procedures, RVE^34^ and REML,^35^ each paired with six variance formulas used to calculate the ICC weights.

### 3.3 Analytic methods

#### 3.3.1 Meta-analytic methods

For each meta-analytic dataset of ICC estimates, we compared random-effects pooling using REML^35^ with random-effects pooling using RVE^34^ when estimating the average pooled ICC estimate.

All simulations were conducted in R.^72^ We used the robumeta package^73^ to implement the RVE model using the robu() function with the small-sample corrections for both the residuals and degrees of freedom.^60^ We used the metafor package^57^ to fit the random effects model with REML estimation by setting the “method” argument in the rma.uni() function to “REML” and the test argument set to “knha” for Knapp and Hartung test.^74^

#### 3.3.2 Variance of the ICC estimate

We compared derivations of the variance for the ICC estimate when used as inverse variance weights in the meta-analytic pooling methods. We compared the formulas derived by Donner and Koval,^52^ Hedges et al.,^27^ Fisher,^1^ Swiger et al.,^26^ Smith,^2^ and Fisher^29^ which are Equations (2.4)–(2.10) in the previous section. We coded functions for each of these variance formulas in R. We used the lmeInfo package^75^ to extract the variance of the level-2 variance ( $v_{2}$ ) because the formula proposed by Hedges et al.^27^ (Equation (2.6)) requires it.

For Equation (2.10), additional steps were taken. First, we transformed the ICC estimates using Equation (2.9) before implementing the meta-analytic pooling methods. We used the variance formulation presented by Fisher^29^ only for the transformed ICC estimates. Then, we back-transformed the overall pooled estimate using the arithmetic average of the weighted mean cluster sizes from the meta-analytic dataset used to estimate the pooled ICC estimate.

### 3.4 Performance criteria

Because we aimed to determine how well each variance of the ICC and meta-analytic method recovers the value of the ICC parameter used to generate the raw data, we captured the difference using the parameter bias (PB) specified as

(3.1) $$\begin{array}{r} PB(\hat{\rho })=\overset{―}{\hat{\rho }}-\rho , \end{array}$$

where the PB is the difference between the mean of pooled ICC estimates across all replications ( $\overset{―}{\hat{\rho }}$ ) and the true generating value of ICC. The corresponding MCSE is $MCSE_{PB}=\sqrt{S_{\hat{\rho }}^{2}/K}$ , where *K* is the number estimates and $S_{\hat{\rho }}^{2}$ is the sample variance: $S_{\hat{\rho }}^{2}=\frac{1}{K-1}\sum_{k=1}^{K}({\hat{\rho }}_{k}-\overset{―}{\hat{\rho }}{)}^{2}$ .

Furthermore, we also captured the difference using relative PB (RPB) specified as

(3.2) $$\begin{array}{r} RPB(\hat{\rho })=\frac{\overset{―}{\hat{\rho }}-\rho }{\rho }. \end{array}$$

If the value of the RPB is less than |0.05| , then that is considered an acceptable level of bias.^76^ We used RPB to complement the use of PB. RPB contextualizes the degree of PB relative to the scale of the parameter’s true value. RPB also offers some criteria that some researchers use to identify “substantial” versus “not substantial” bias.^76^ However, sometimes the scale of the true parameter value can inflate the RPB. Thus, we looked at both PB and RPB.

We also calculated MCSE as: $MCSE_{RPB}=(1/\rho )\sqrt{S_{\hat{\rho }}^{2}/K}$ . Furthermore, we captured the RPB of each random-effects variance component used to calculate the ICC, as seen in Equation (2.2), in order to determine if any source of bias in the pooled ICCs might be due to poorly estimated ICCs from the raw data.

We computed the root mean square error (RMSE) to measure the accuracy of the resulting estimators. RMSE evaluates the proximity of the average pooled ICC estimate to the true generating value of ICC and the precision of the estimation. The smaller the RMSE value, the better. For the true value of the ICC parameter, ρ , and its estimate, $\hat{\rho }$ , RMSE is

(3.3) $$\begin{array}{r} RMSE(\hat{\rho })=\sqrt{\frac{1}{K}\sum_{k=1}^{K}({\hat{\rho }}_{k}-\rho )}. \end{array}$$

The primary motivation for this study was to assist researchers needing to impute an accurate pooled ICC estimate to use in a power analysis for clustered data or to correct effect sizes for clustering in primary study data used in a meta-analysis. SE estimates for the imputed ICC in these scenarios are not used. However, for researchers who intend to meta-analyze ICC estimates, we also evaluated which ICC pooling method results in the best SE estimate for the pooled ICC estimate. We also captured the relative SE bias (RSEB) of the SE for the pooled ICC estimate. We expect to see differences in the SEs of the pooled ICC estimate when using different estimation procedures. We calculated RSEB using

(3.4) $$\begin{array}{r} RSEB(SE_{\hat{\rho }})=\frac{{\overset{―}{SE}}_{\hat{\rho }}-S_{\hat{\rho }}}{S_{\hat{\rho }}}, \end{array}$$

where ${\overset{―}{SE}}_{\hat{\rho }}$ is the mean of the SE estimates and $S_{\hat{\rho }}$ is the standard deviation of the pooled ICC estimates across replications.

## 4 Results

### 4.1 Simulation study results

**Figure 3:**
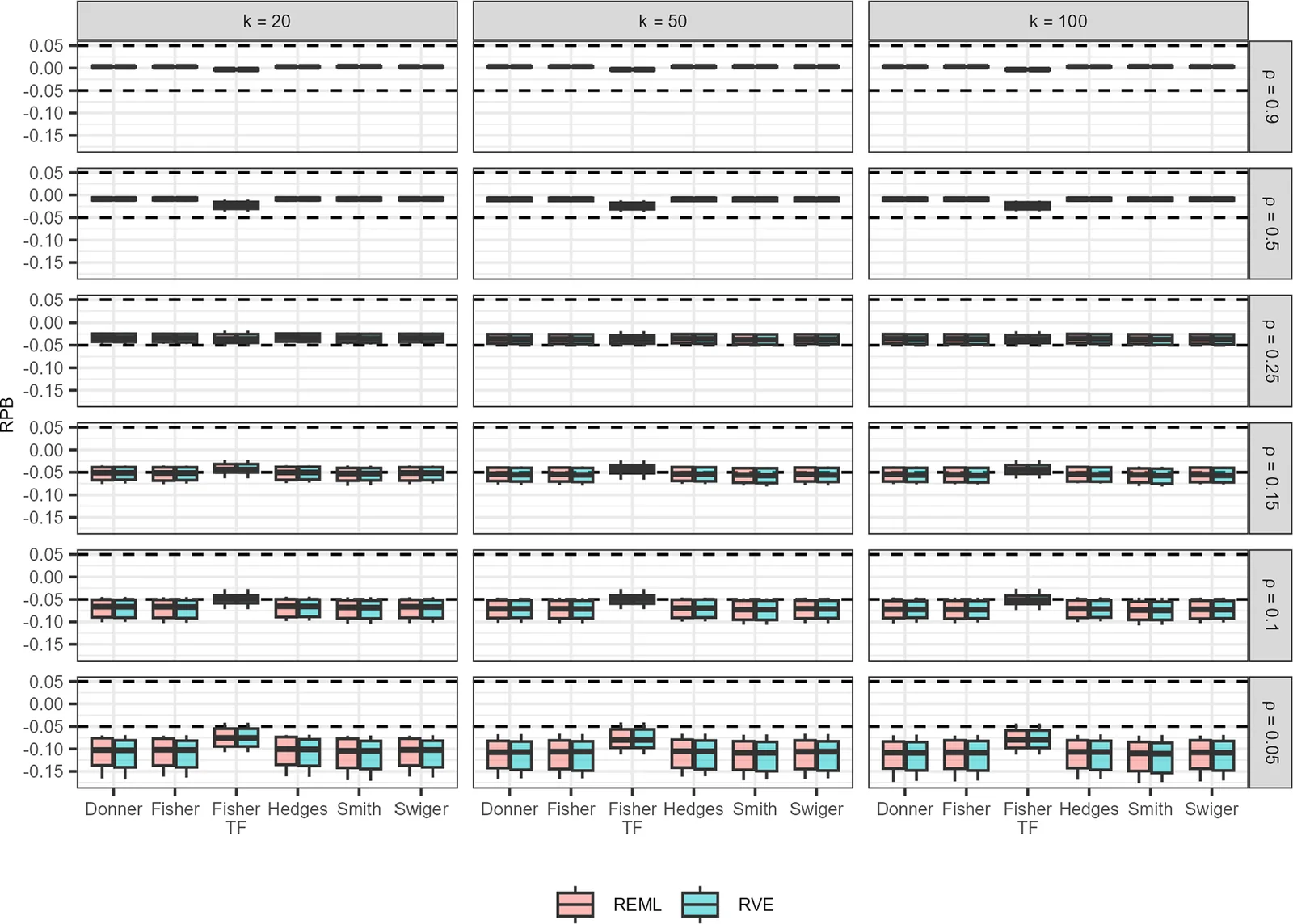
The RPB of the pooled ICC estimates by the method of pooling, the number of ICC estimates pooled (*k*), and the generating true ICC ( ρ ). Dashed lines mark boundaries of acceptable level of bias ( ±0.05 ). Figure 3 Long description.

**Figure 4:**
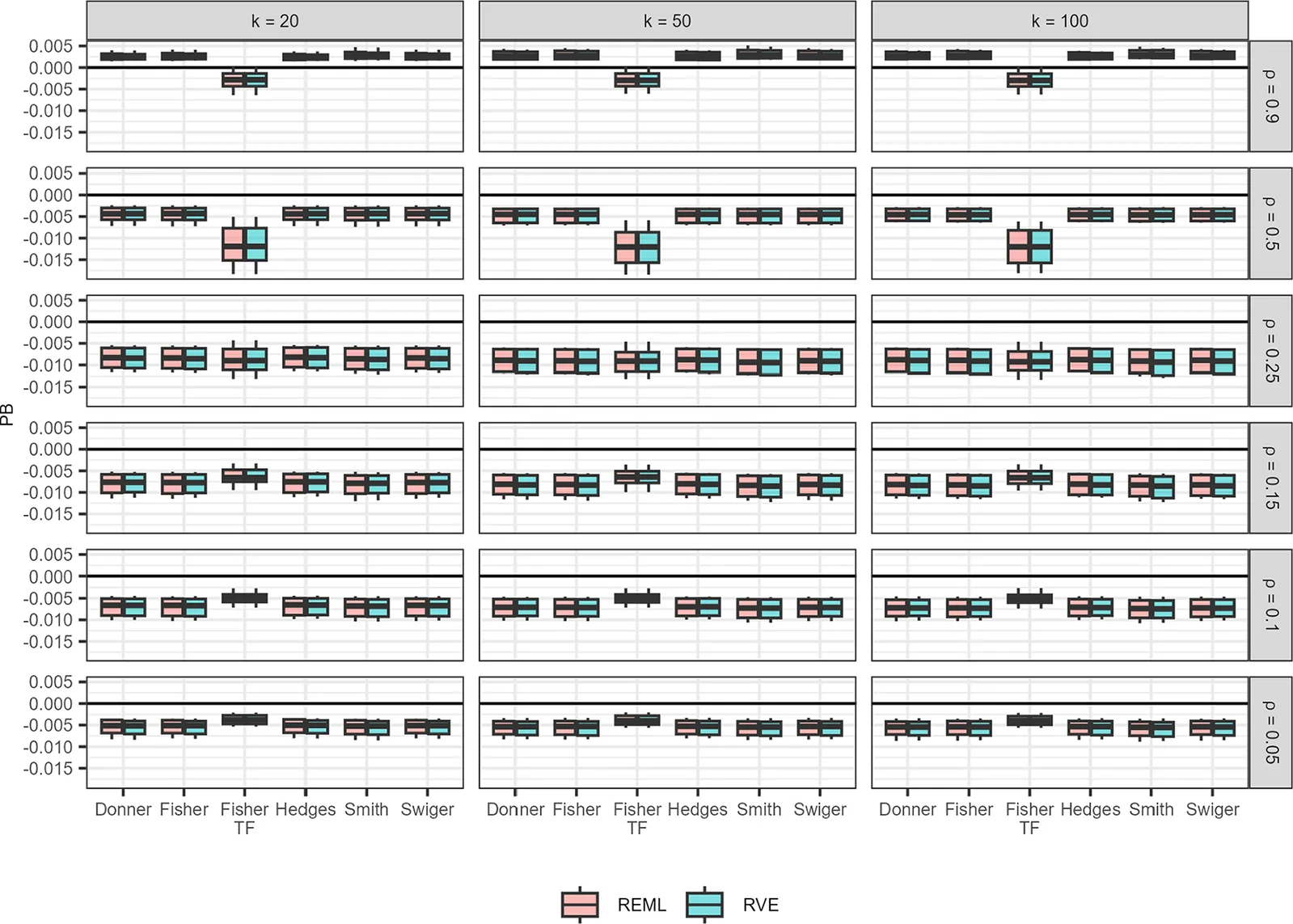
The PB of the pooled ICC estimates by the method of pooling, the number of ICC estimates pooled (*k*), and the generating true ICC ( ρ ). Solid line is at 0. Figure 4 Long description.

**Figure 5:**
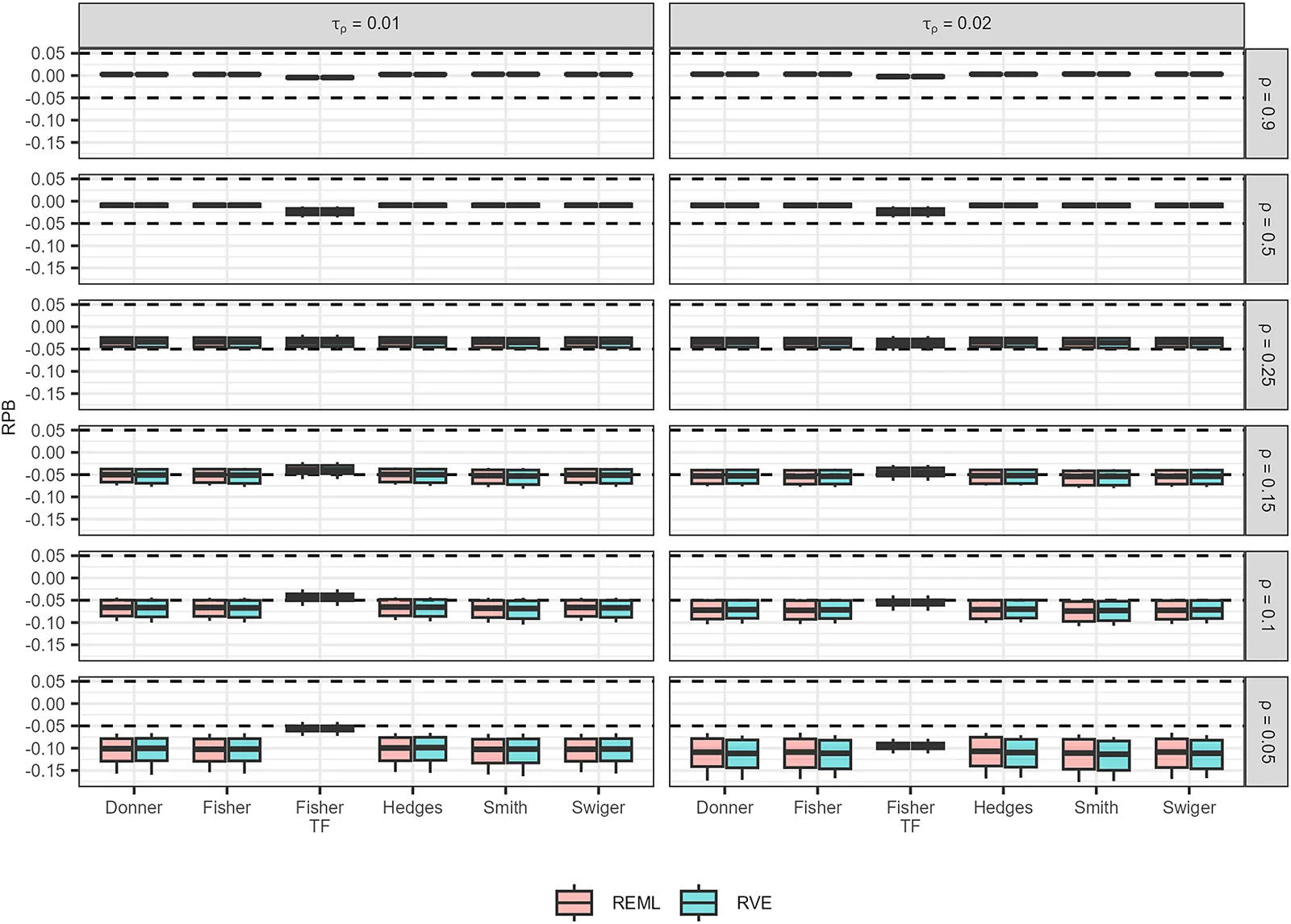
The RPB of the pooled ICC estimates by the method of pooling, the between-study heterogeneity ( $\tau_{\rho }$ ), and the generating true ICC ( ρ ). Dashed lines mark boundaries of acceptable level of bias ( ±0.05 ). Figure 5 Long description.

**Figure 6:**
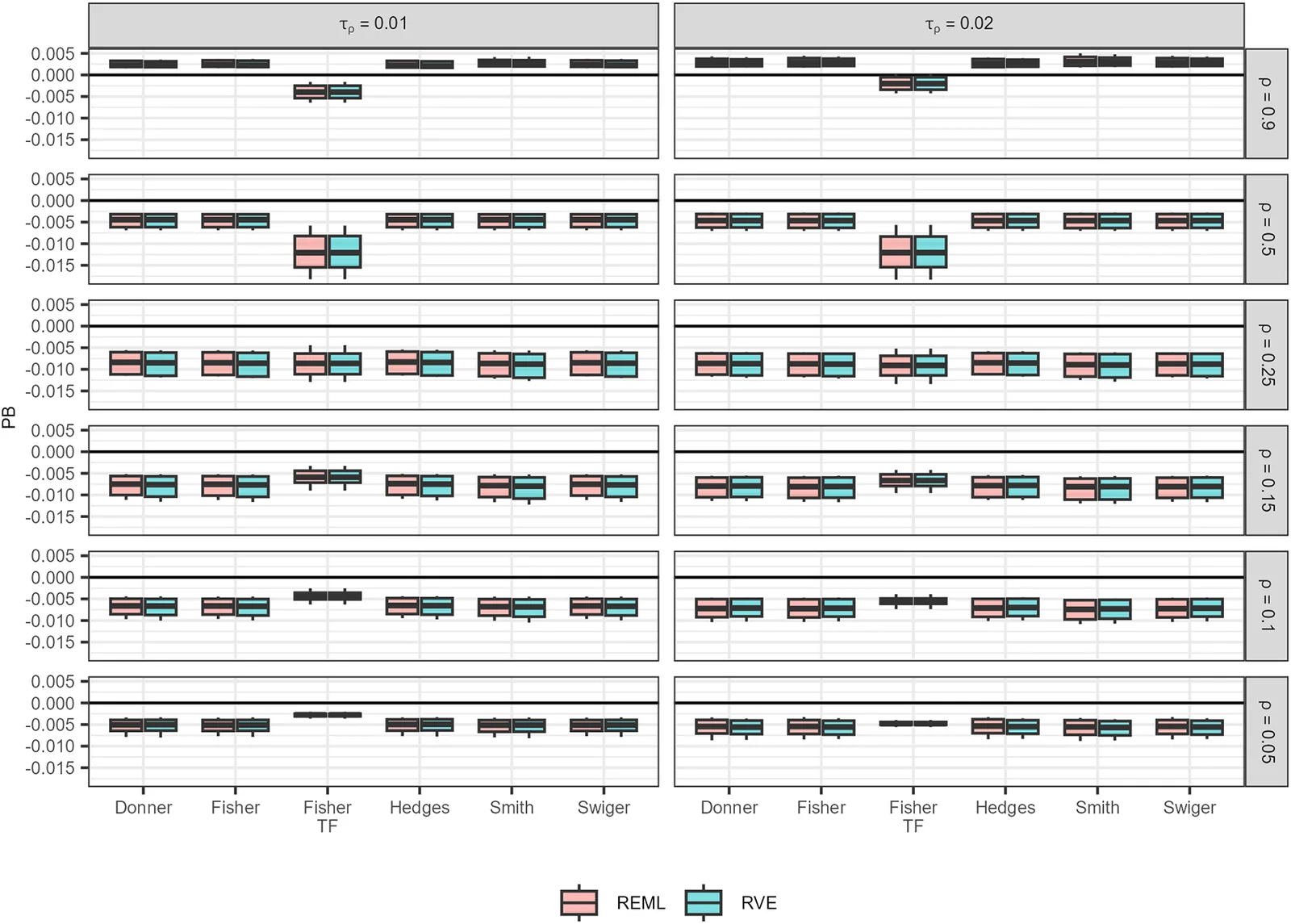
The PB of the pooled ICC estimates by the method of pooling, the between-study heterogeneity ( $\tau_{\rho }$ ), and the generating true ICC ( ρ ). Solid line is at 0. Figure 6 Long description.

**Figure 7:**
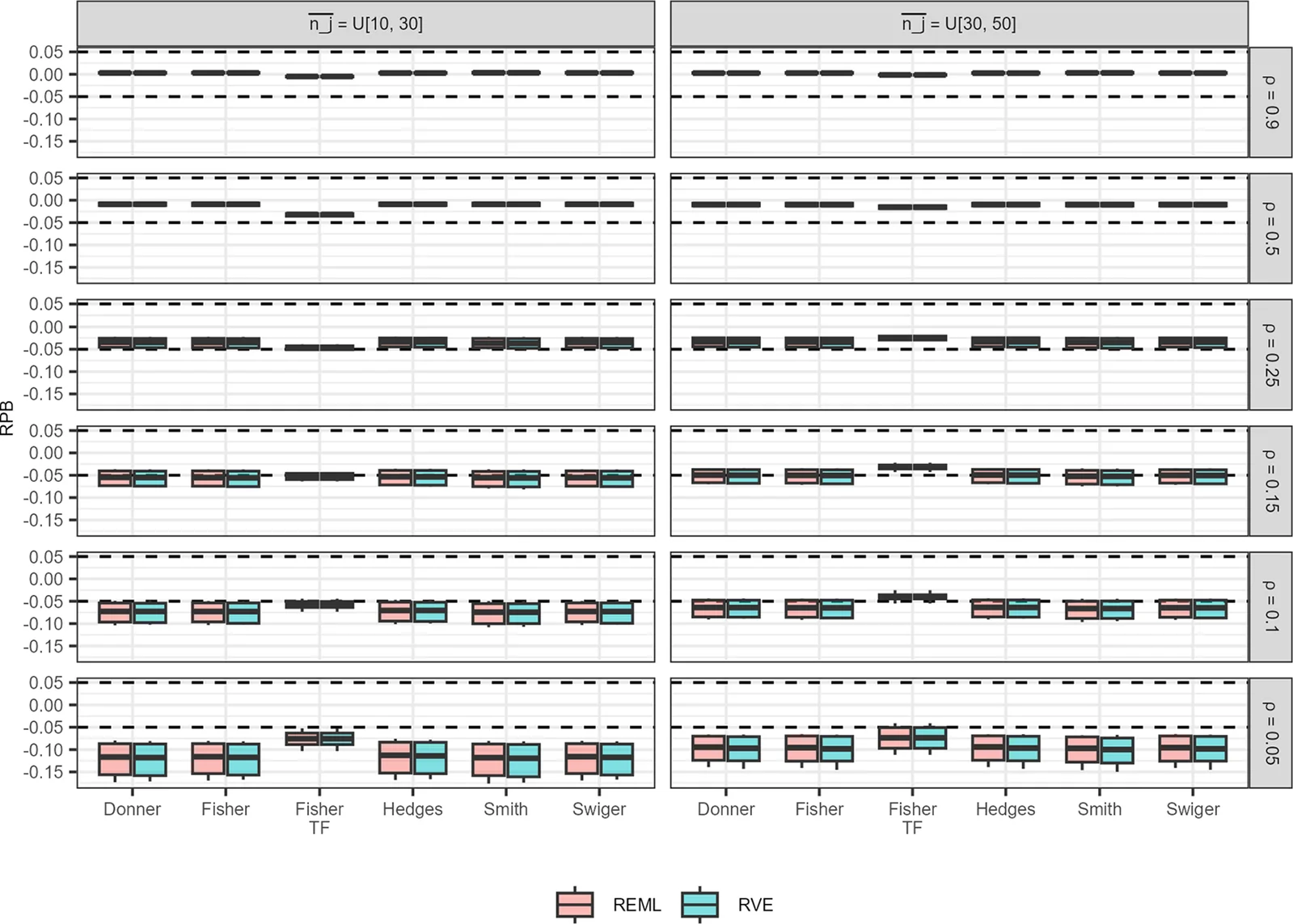
The RPB of the pooled ICC estimates by the method of pooling, the average number of units per cluster ( ${\overset{―}{n}}_{j}$ ), and the generating true ICC ( ρ ). Dashed lines mark boundaries of acceptable level of bias ( ±0.05 ). Figure 7 Long description.

**Figure 8:**
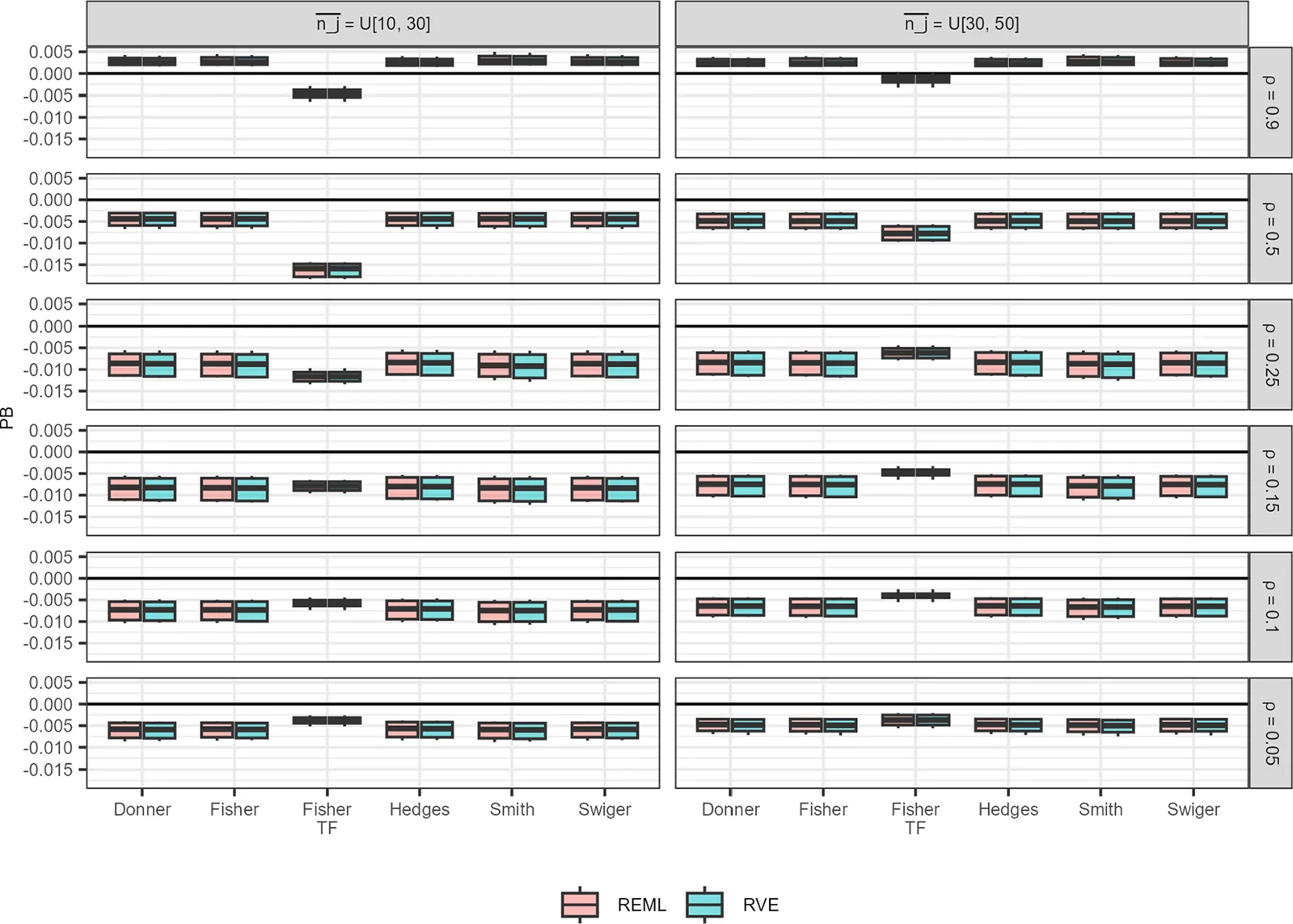
The PB of the pooled ICC estimates by the method of pooling, the average number of units per cluster ( ${\overset{―}{n}}_{j}$ ), and the generating true ICC ( ρ ). Solid line is at 0. Figure 8 Long description.

**Figure 9:**
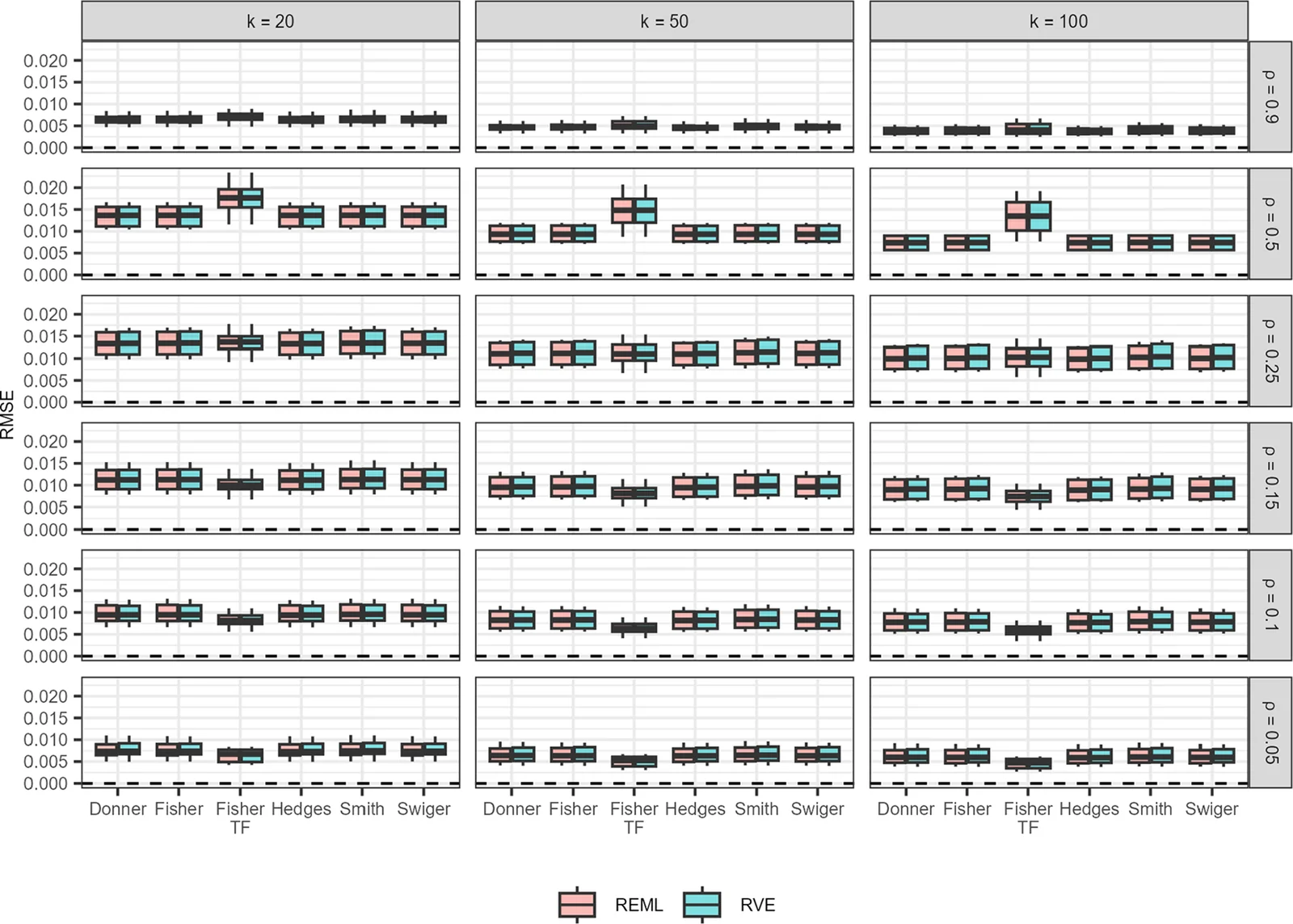
The RMSE of the pooled ICC estimates by the method of pooling, the number of ICC estimates pooled (*k*), and the generating true ICC ( ρ ). Dashed line is at 0. Figure 9 Long description.

**Figure 10:**
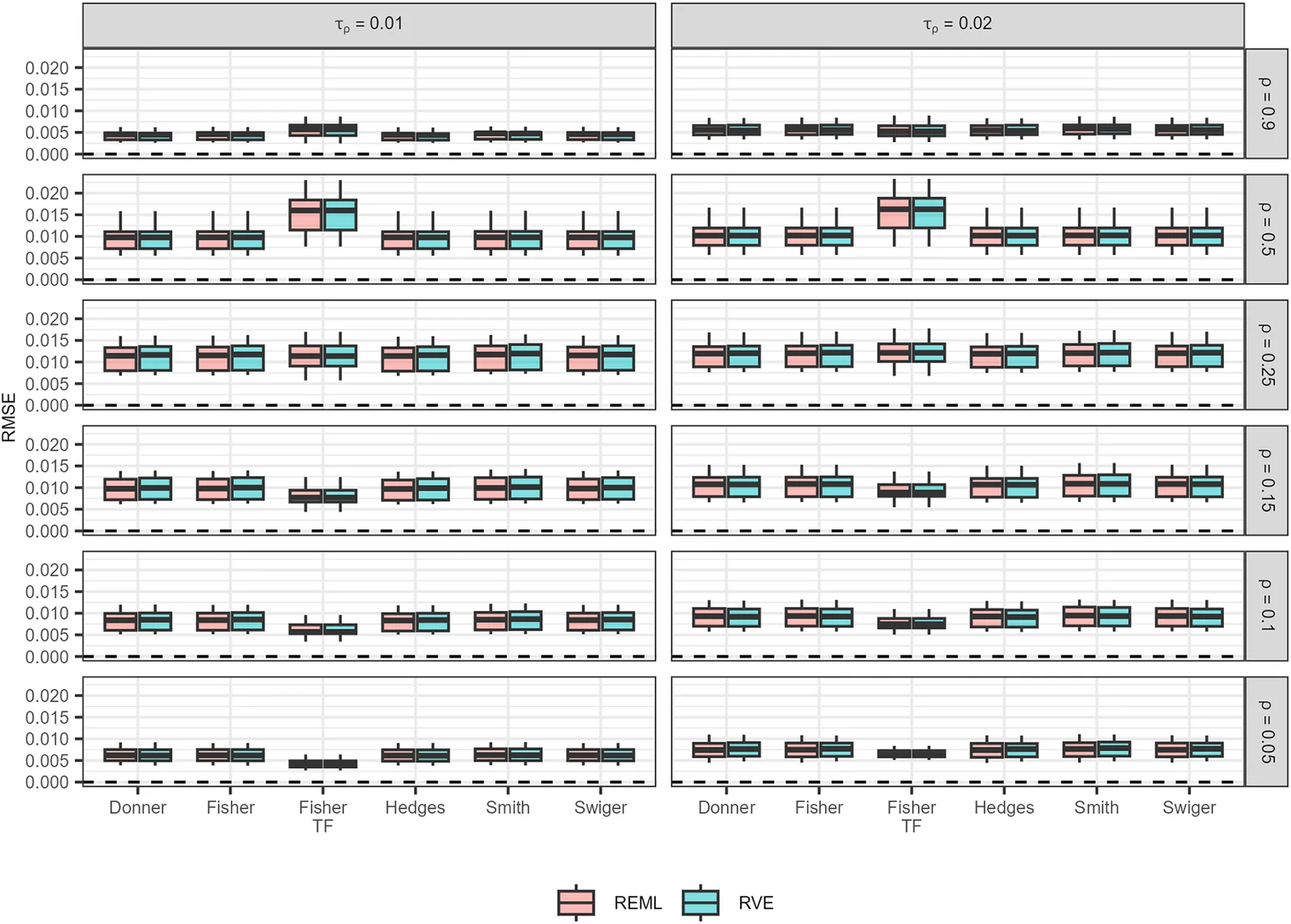
The RMSE of the pooled ICC estimates by the method of pooling, the between-study heterogeneity ( $\tau_{\rho }$ ), and the generating true ICC ( ρ ). Dashed line is at 0. Figure 10 Long description.

**Figure 11:**
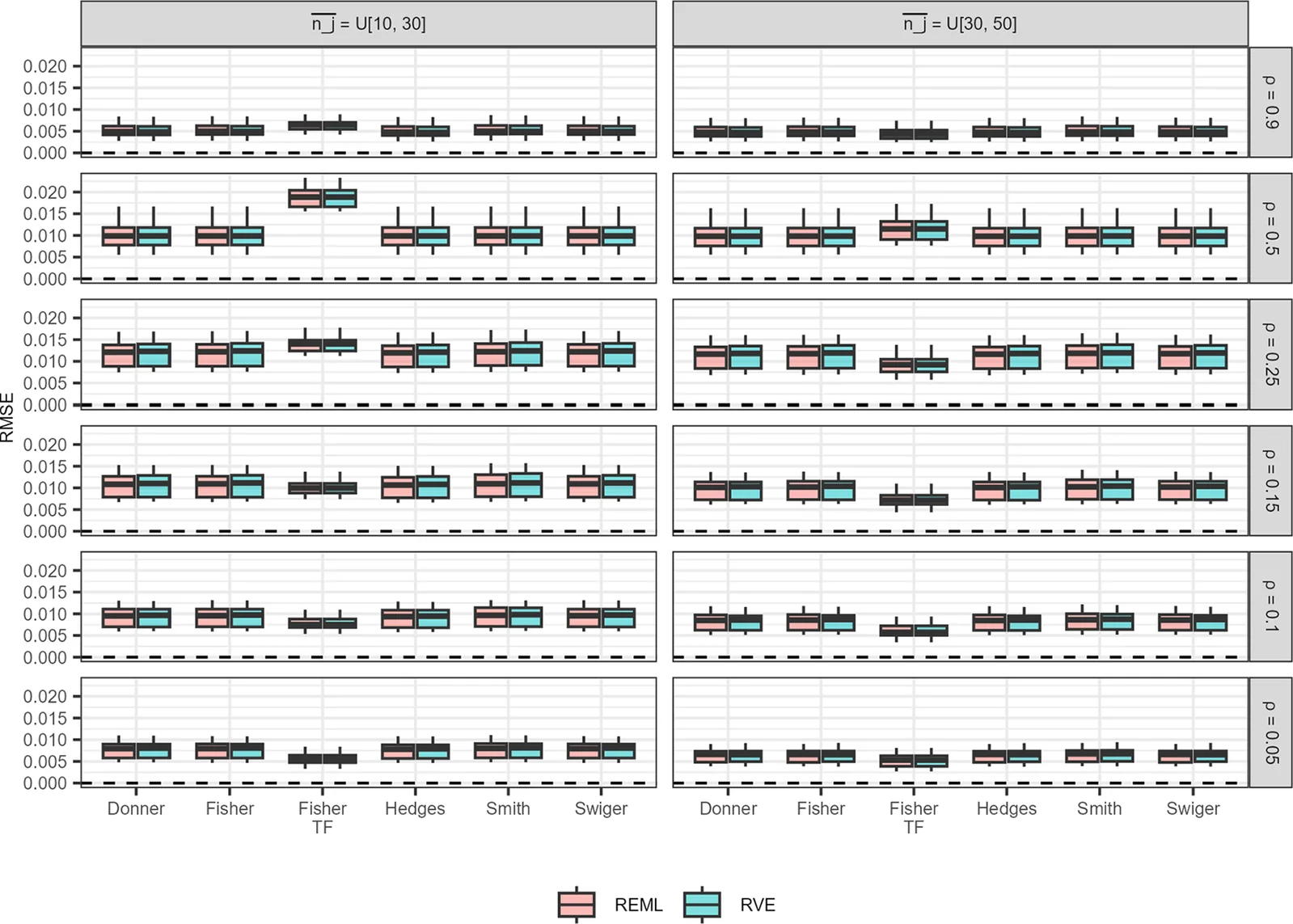
The RMSE of the pooled ICC estimates by the method of pooling, the average number of units per cluster ( ${\overset{―}{n}}_{j}$ ), and the generating true ICC ( ρ ). Dashed line is at 0. Figure 11 Long description.

**Figure 12:**
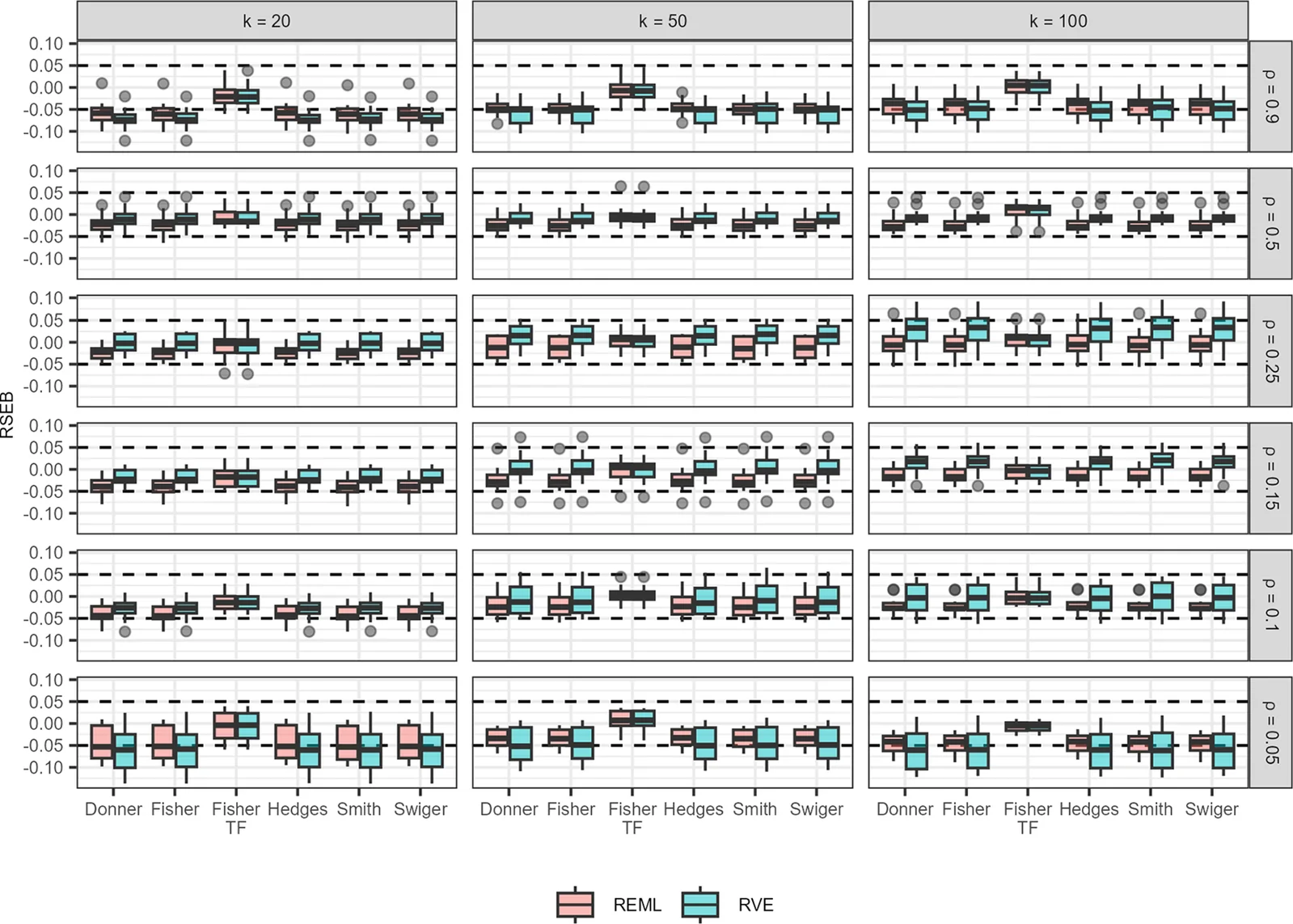
The RSEB of the pooled ICC estimates by the method of pooling, the number of ICC estimates pooled (*k*), and the generating true ICC ( ρ ). Dashed lines mark boundaries of acceptable level of bias ( ±0.05 ). Figure 12 Long description.

**Figure 13:**
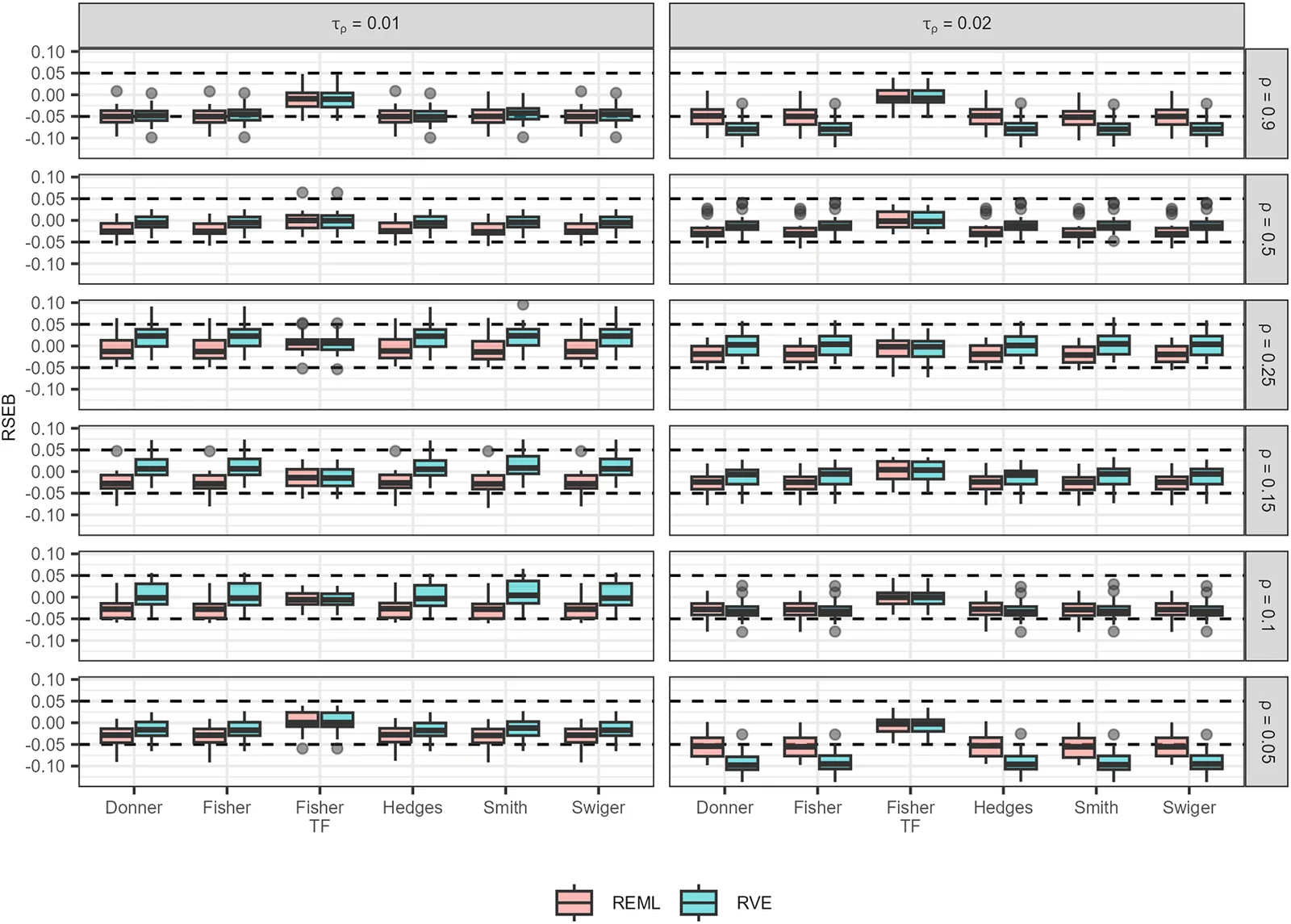
The RSEB of the pooled ICC estimates by the method of pooling, the between-study heterogeneity ( $\tau_{\rho }$ ), and the generating true ICC ( ρ ). Dashed lines mark boundaries of acceptable level of bias ( ±0.05 ). Figure 13 Long description.

**Figure 14:**
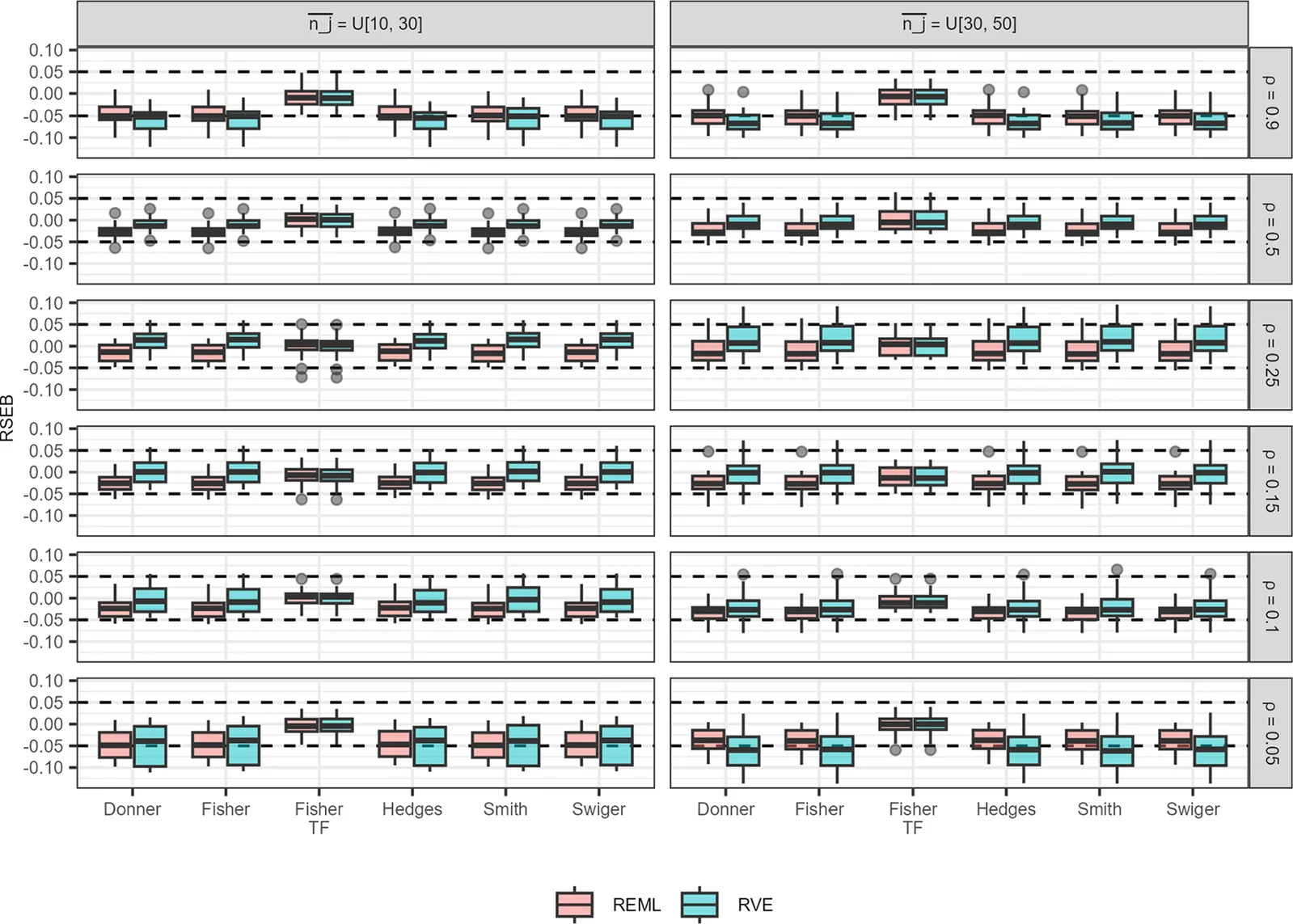
The RSEB of the pooled ICC estimates by the method of pooling, the average number of units per cluster ( ${\overset{―}{n}}_{j}$ ), and the generating true ICC ( ρ ). Dashed lines mark boundaries of acceptable level of bias ( ±0.05 ). Figure 14 Long description.

**Figure 15:**
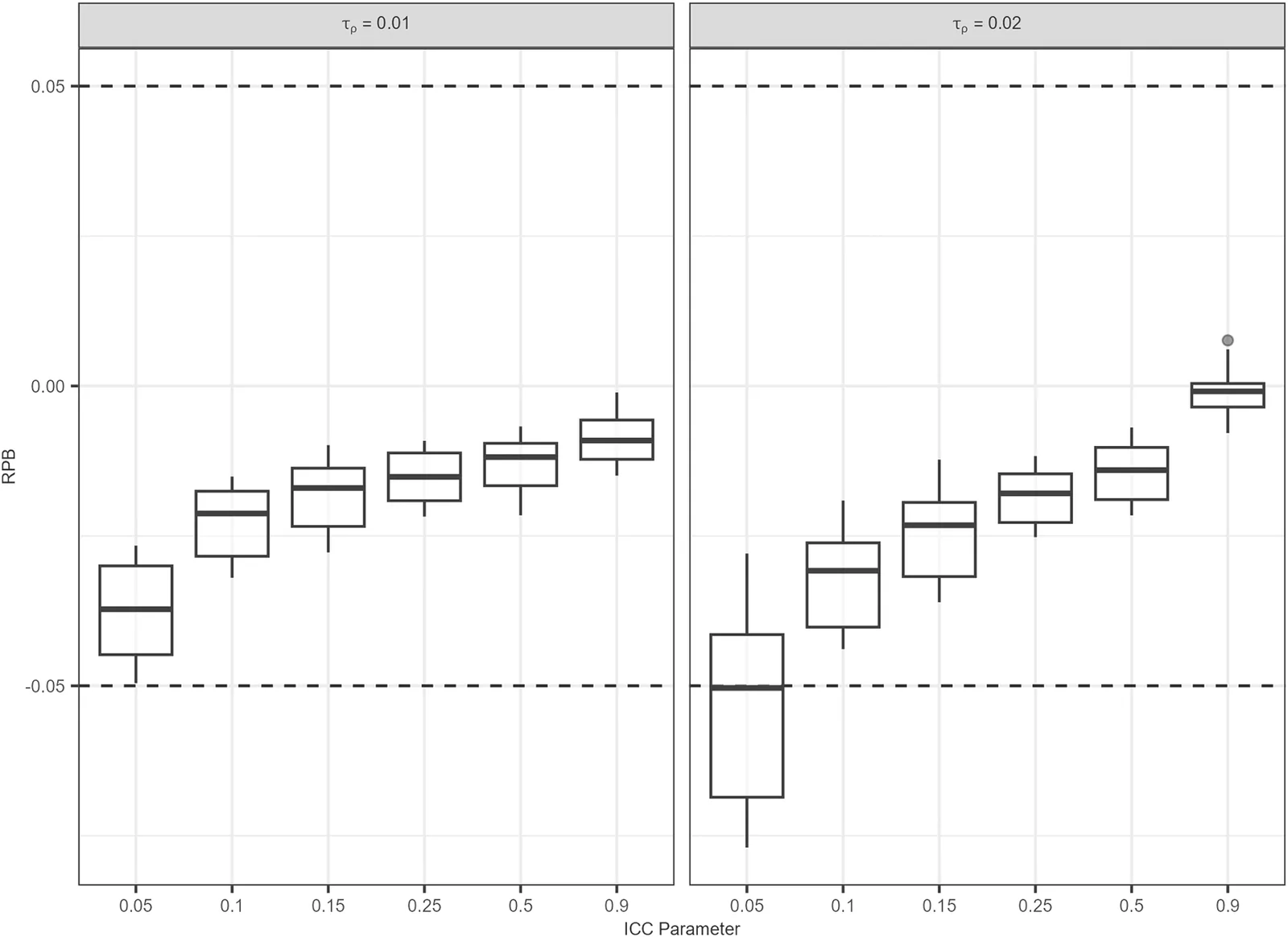
The RPB of the level-2 variance estimate by the between-study heterogeneity ( $\tau_{\rho }$ ). Dashed lines mark boundaries of acceptable level of bias ( ±0.05 ). Figure 15 Long description.

**Figure 16:**
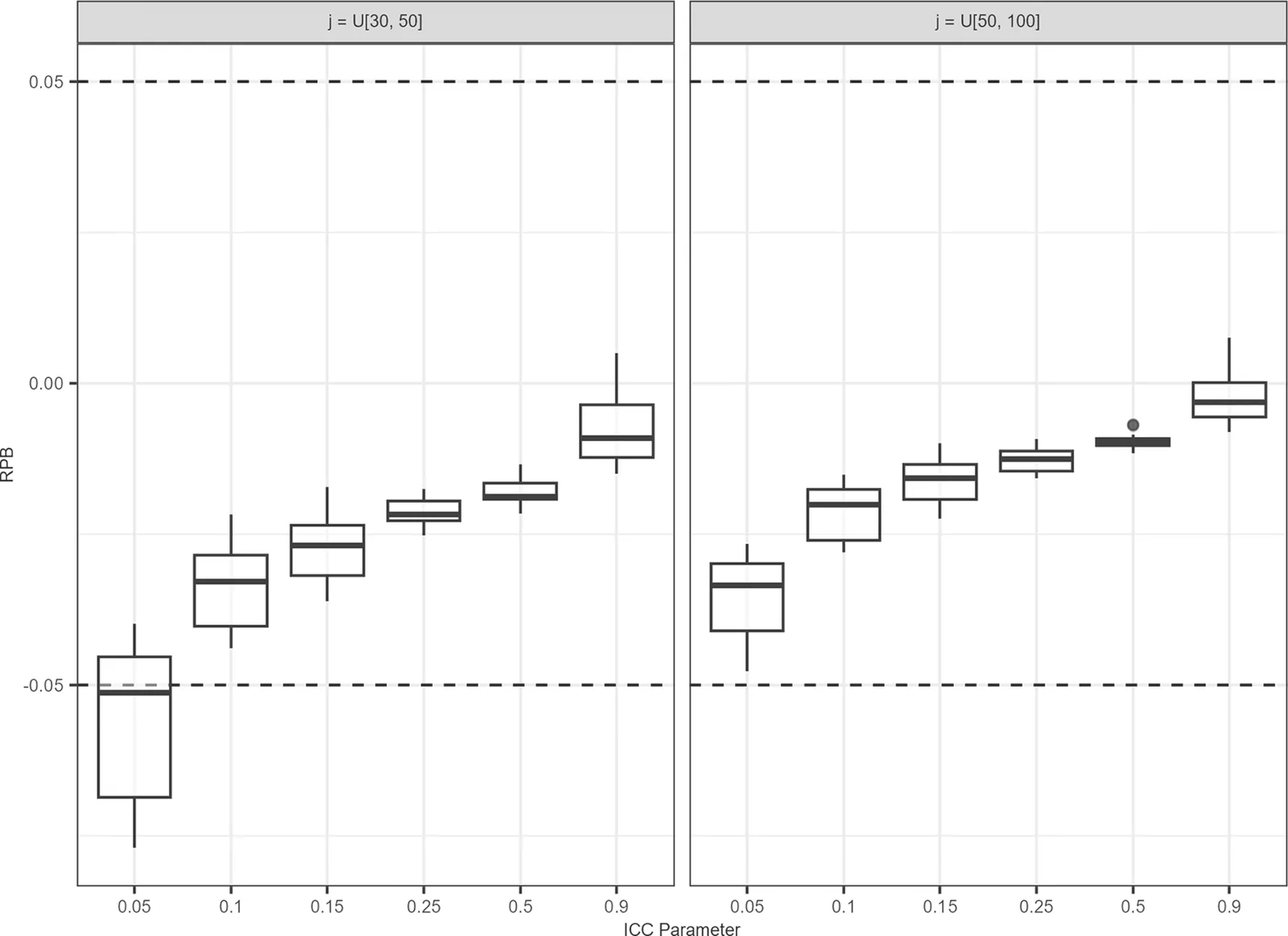
The RPB of the level-2 variance estimate by the number of clusters (*j*). Dashed lines mark boundaries of acceptable level of bias ( ±0.05 ). Figure 16 Long description.

In this section, we present the salient results of the performance of the different estimation methods when paired with the different variance formulas in the weight formula for the pooled ICC estimate, SE of the pooled ICC estimate, and the level-2 variance ( $\sigma_{u}^{2}$ ) when examining combinations of the manipulated conditions we included in the simulation. The Monte Carlo SEs of bias and RPB were all less than 0.01 across all conditions. Below, in Figures 3–11, we first present the bias, RPB, and root mean squared error results for the pooled ICC estimate. Then, in Figures 12–14, we present the results of the RSEB for the SE of the pooled ICC estimate. Finally, in Figures 15 and 16, we focus on the RPB of the level-2 variance estimate.

REML estimation, paired with all the variance formulas (except Fisher TF), had occasional issues converging across conditions. We ran 1,500 replications of each condition to ensure that we captured results from analysis of at least 1,000 converged replications per method. Across all 1,500 replications, most of the non-convergence for REML occurred in the small number of clusters condition ( U [30, 50]). The lowest convergence rate was 99.13% across conditions. To compare the same number of replication datasets for each condition, the graphs only summarize the same set of 1,000 replications per condition.

All the results from this section and supplementary analyses, a table summarizing the average, variance, and range of the performance criteria for each method, and the R code for replicating the study are available on the Open Science Framework at https://osf.io/g5npu.

#### 4.1.1 Pooled ICC estimate

Figure 3 presents the RPB estimates as a function of the number of estimates being pooled (*k*) and the true generating ICC values ( ρ ). This figure shows that there are no differences between using REML and RVE in the overall pooled estimate when pooling ICC estimates across the conditions we evaluated. Furthermore, we found no differences as a function of the number of ICC estimates pooled using 20, 50, or 100 estimates. However, the Fisher formula for $v_{\hat{\rho }}$ that uses a normalizing transformation of ρ (Fisher TF; Equation (2.9)) performs best across the different $v_{\hat{\rho }}$ formulas when used as the inverse weight formula. For larger ρ (e.g., >0.25 ), the $v_{\hat{\rho }}$ formulas did not matter, and all formulas resulted in unbiased estimates for RPB. However, we did not find the same result for PB (Figure 4); while when ρ=0.25, there were virtually no differences between the $v_{\hat{\rho }}$ formulas, when ρ=0.5 , we found that the Fisher TF formula resulted in more negative raw bias than the other variance formulas, but the magnitude of the bias was very small with it at most resulting in a bias of approximately −0.018 for both REML and RVE versus about −0.007 for the other variance formulas for REML and RVE. For ρ=0.9 , there were no substantial differences among the results for the variance formulas, but the Fisher TF formula did result in a slightly underestimated pooled ICC estimate (with the most bias being −0.006 for both REML and RVE), while using the other variance formulas slightly overestimated pooled ICC estimates (by at most 0.0017 with the Smith formula).

Figure 5 displays the RPB estimates as a function of between-study heterogeneity ( $\tau_{\rho }$ ) and the true generating ICC values ( ρ ). There were very small differences as a function of the degree of between-study heterogeneity that we evaluated. Higher between-study heterogeneity when the true generating ICC values are small ( ρ=0.05 ) resulted in negative RPB estimates across all methods, with the Fisher TF variance formula resulting in the least biased estimates. For the ρ values of 0.15, the estimates were very close to lacking substantial RPB, and no substantial RPB was found when ρ was generated using values of ρ≥0.25 . Figure 6 displays the PB estimates as a function of between-study heterogeneity ( $\tau_{\rho }$ ) and the true generating ICC values ( ρ ). The same pattern is shown here, where when ρ=0.05 , the higher between-study heterogeneity condition resulted in more negative bias across conditions. Also, we see that between-study heterogeneity does not explain why the Fisher TF formula does somewhat worse than the other formulas when ρ=0.5 .

Figures 7 and 8 display the results for the RPB and PB, respectively, with the column facets as the average cluster size and the row facets as the generating ρ . There is a non-linear interaction between the magnitude of ICC and the average cluster size in their effects on PB. For the other variance formulas, the cluster size does not seem to impact the bias, but for the Fisher TF formula, particularly when the ρ values are in the middle range ( ρ=0.25 or ρ=0.5 ), when the average cluster size of the primary studies that comprise a meta-analytic dataset were drawn from smaller values $n_{j}∼U[10,30]$ ), this resulted in more biased pooled ICC estimates for Fisher TF compared to when they were drawn from large values ( $n_{j}∼U[30,50]$ ).

Figure 9 provides the results for the RMSE of the pooled ICC estimate as a function of the number of estimates being pooled (*k*) and the true generating ICC values ( ρ ). Consistent with the PB results, the Fisher TF variance formula results in the lowest RMSE for small true generating ICC values ( ρ=0.05 and ρ=0.15 ), but the highest RMSE when the true generating ICC value was 0.5 .

Figure 10 contains the results for the RMSE of the pooled ICC estimate as a function of between-study heterogeneity ( $\tau_{\rho }$ ) and the true generating ICC values ( ρ ). The degree of between-study heterogeneity did not explain any of the differences in RMSE across the various sampling variance formulas.

Figure 11 contains the results for the RMSE of the pooled ICC estimate as a function of average cluster size ( $\overset{―}{n_{j}}$ ) and the true generating ICC values ( ρ ). The graph shows that when ρ=0.5 , the Fisher TF formula results in worse RMSE values than the other formulas when the average cluster sizes were drawn from U[10,30] . However, we found that Fisher’s TF generally has lower RMSE values (or the same) across all values of ρ when the average cluster sizes are larger (i.e., drawn from U[30,50] ).

#### 4.1.2 SE of the pooled ICC estimate

Figure 12 displays results for the RSEB of the pooled ICC estimate as a function of the number of estimates being pooled (*k*) and the true generating ICC values ( ρ ). In addition, the number of ICC estimates pooled does not make a difference in RSEB across the conditions we evaluated, except in the ρ=0.05 condition. For the ρ=0.05 , there are differences in performance between RVE and REML. Surprisingly, the larger the number of ICC estimates, the worse the pooled estimates are for RVE compared to REML in the ρ=0.05 condition, except with Fisher’s TF variance formula. Figure 13 displays the results for RSEB of the pooled ICC estimates by between-study heterogeneity ( $\tau_{\rho }$ ) and the true generating ICC value. When the true generating ICC is small ( ρ=0.05 ), the degree of heterogeneity does affect the performance of RVE, where with a larger $\tau_{\rho }$ value, it results in more negative RSEB values compared to REML, but when $\tau_{\rho }$ is smaller, there is no difference between the methods in this condition.

Finally, Figure 14 has the results for the RSEB of the pooled ICC estimates, average cluster size ( ${\overset{―}{n}}_{j}$ ), and the generating true ICC ( ρ ). Despite the average cluster size having an impact on the pooled ICC estimate for the Fisher TF formula, it does not impact the SE of the pooled ICC estimate, and it performs the best among the rest of the variance formulas.

#### 4.1.3 Level-2 variance of primary studies

Figures 15 and 16 display results for the RPB of the level-2 variance. Figure 15 provides a box plot of level-2 variance estimates by between-study heterogeneity. As shown in the figure, for $\tau_{\rho }=0.02$ and ρ=0.05 , the level-2 variance estimates were slightly biased when the generating true ICC was small ( ρ=0.05 ). We also wanted to confirm that the level-2 variance of the primary study recovered well across the number of clusters as can be seen in Figure 16. When the number of clusters was drawn from U(30,50) and ρ=0.05 , the resulting level-2 variance estimates were slightly biased. Otherwise, estimation of the level-2 variance was not substantially biased across conditions.

## 5 Discussion

There is a need for using accurate ICC values for a priori power analyses or meta-analyses of effect sizes in clustered data scenarios. The most frequently used methods for imputing ICC estimates in these scenarios can result in inaccurate values that ultimately negatively impact the inferences from the associated analyses in which the values are used. Little research has focused on capturing reasonable ICC values to improve the validity of associated inferences. Meta-analytic pooling of relevant observed ICC values offers a principled method for imputing ICC values. However, there has not been much research exploring how best to meta-analyze ICC estimates, despite ICC estimates already being relatively regularly meta-analyzed, particularly in the reliability literature. The unknown shape of the sampling distribution of ICC estimates offers a particular challenge to the meta-analytic pooling of ICC estimates. In addition, the formulation for the variance of the sampling distribution of ICC estimates is unknown and the shape of the empirical distribution varies depending on the magnitude of the ICC and the data context (ICCs for clustered individuals or for clustered measurements), although several methodological researchers have suggested various formulas.^1^ ^,^ ^2^ ^,^ ^26^ ^,^ ^27^ ^,^ ^29^ ^,^ ^52^ The current study compares approximations of the sample variance formulas (used in the weighting of ICCs when they are pooled) and compares two methods for meta-analytic pooling (REML versus RVE) across a number of design conditions. Ultimately, this study is intended to assess the meta-analytic pooling of ICCs to inform researchers about the best way to do so.

The results of this study provide some guidelines for applied researchers when (1) they need to impute ICC values for use in an a priori power analysis for clustered data, (2) the researcher is conducting a meta-analysis that contains effect sizes for clustered data in any of the primary studies in the meta-analytic dataset that need to be corrected for clustering or measurement error and that might be missing associated ICC estimates, or (3) the researcher is meta-analyzing ICC estimates. The results suggest that, for the design conditions we examined, there were no considerable differences in the pooled ICC estimates when using REML versus using RVE to pool ICC estimates. Therefore, when meta-analyzing ICCs in the kinds of scenarios that we examined, REML and RVE appear to function equivalently. There were no huge differences among variance formulas, but the Fisher TF formula (Equation (2.10); Fisher^29^) performed best across the design conditions we tested for RPB. The Fisher TF formula provided the least biased pooled ICC estimates and SE of the ICC estimates for the ρ=0.05 conditions, and it was the only $v_{\hat{\rho }}$ formula without substantial bias in the pooled ICC estimates in the ρ=0.15 conditions. For PB difference and when ρ=0.5 , the Fisher TF formula resulted in smaller pooled ICC estimates compared to the other formulas when the average cluster size was small (10–30 range), but the degree of bias was small with at most the maximum bias observed having a value of −0.018 from the true ICC parameter of 0.5 . In addition, Fisher’s TF results were still comparable with results from other formulas in that condition. Furthermore, it seemed that the average cluster size explained this interaction. This is probably due to the formula only requiring the weighted mean cluster size and the ICC estimate in its formulation, unlike the other formulas. For the RSEB, it is not clear why there is a mixed pattern as a function of *k* and ρ , but even in this, the Fisher TF formula performs best. Furthermore, we do see that RVE results in more biased RSEB values when ρ=0.05 or ρ=0.9 when there is a higher degree of between-study heterogeneity ( $\tau_{\rho }=0.02$ ), with RVE being particularly impacted in those conditions. RMSE is influenced by bias and variability. For the RMSE of the pooled ICC estimate, the bias alone explained the result for the Fisher TF formula, having the largest RMSE value when ρ=0.5 . Furthermore, for the purposes of imputing an accurate ICC estimate in the context of power analysis of clustered data or correcting effect sizes for clustering, estimating an SE is not necessary. Across all conditions, the Fisher TF formula never resulted in substantially biased pooled estimates and associated SEs, which was not true for any of the other variance formulas examined here. So, we recommend using the Fisher TF formula for the ICC variance for use in the inverse variance weights when pooling ICC estimates under the conditions we evaluated.

Regarding primary study characteristics, larger sample sizes resulted in less biased pooled ICC estimates across all $v_{\hat{\rho }}$ formulas. Last, we did not find any distinctive impact on the pooled ICC estimation of the number of ICC estimates being pooled ( k= 20, 50, versus 100). Future studies could evaluate how well pooling fewer ICC estimates captures the true ICC parameter value.

Finally, these meta-analytic pooling ICC estimates methods are relatively accessible to applied researchers. For example, RVE for meta-analysis is available in software like Stata, SPSS, and R. These pooling methods are already being used widely by applied meta-analysts.

For this study, we needed to consider simulation design factors in generating primary study data to estimate an ICC estimate, when generating a meta-analytic dataset of ICC estimates, and when assuming a distribution for the population of ICCs. Due to all these factors, we needed to limit the simulation design, which limits our findings. There are many ways that this study could be extended.

The following are the limitations of the simulation design conditions we chose that directly impact the generation of an individual ICC estimate. We simulated raw primary data assuming that the outcome was normally distributed and continuous, but ICCs can arise from other types of data, and that should be evaluated in future studies. Furthermore, we simulated the ICC meta-analysis data in the context of CRT. For ICC as a reliability measure, the number of measurements within subjects can be much smaller than the one we evaluated. Please see Bonett^77^ on how that might impact the estimation of an ICC. Further work is needed on the impacts of meta-analyzing ICC estimates that arise under those conditions.

We only evaluated the pooling of ICC estimates for ICCs for two-level data, assuming a one-way random effects model. While in educational and social science research, more generally, three-level data are also commonly analyzed. Future research could evaluate the use of these methods with three-level data. Hedges et al.^27^ have derived a formula for the variance of an ICC for clustered data with three levels of nesting. Also, there are other types of ICCs defined beyond one-way random effects,^3^ and further work is needed on meta-analyzing those types of ICCs.

We only estimated the variance components of the primary studies in each simulated meta-analysis using REML. However, researchers use a variety of estimation methods for variance components and do not always report which method they used. As stated before, estimating variance components accurately is essential for getting accurate ICC estimates (see Equation (2.2)), so pooled estimates will only be as accurate as the ICC estimates that are being pooled. Future studies might investigate the impact of various estimation methods for the variance components themselves on which each ICC estimate is based.

Regarding simulation design features that are directly related to the meta-analysis of ICC estimates. For our study, we are examining the combination of a uniform random distribution with an ICC sampling distribution arising from normally distributed primary study data. We were primarily focused on the effect of the sampling error variance of an ICC on pooling ICC estimates. Because we want to be agnostic about the shape of the distribution of ICC parameters, we choose a uniform distribution for the probability distribution of the population distribution of the ICC parameter. Further work is needed to evaluate probability distributions for the population of random effects distribution of ICCs. When focusing on random effect distribution of ICCs, one should look at the Beta distribution since it has the same range as an ICC (0–1) and can take different shapes to account for the different directions of skew. Demetrashvili et al.^54^ used the Beta distribution as the assumed sampling distribution to construct confidence intervals for an ICC estimate for these reasons as well. Furthermore, they also cited a theorem that for two independent variables, $X_{1}$ and $X_{2}$ , that both have a gamma distribution, the following ratio $\frac{X_{1}}{X_{1}+X_{2}}$ will have a Beta distribution^54^ ^,^ ^78^ which is the same form as the ICC.^54^ Furthermore, other values for between-study heterogeneity should be examined (including when there is no between-study heterogeneity, $\tau_{\rho }^{2}=0$ ).

We also investigated scenarios in which each primary study only reported a single ICC estimate. The reality is that studies often report results for multiple outcomes, and therefore, multiple ICC values might be available per primary study. These outcomes may be related, and so will be the resulting ICC estimates. The researcher might want to include all the ICC estimates reported for the related outcomes when pooling ICC estimates. Luckily, RVE is typically used to account for dependence in meta-analytic datasets, so this method should be robust to this limitation. The work accomplished in this study could be extended to evaluate methods for pooling of ICC estimates in scenarios with within-study dependence in the ICCs.

We only examined the performance of the pooled ICC estimate and the corresponding SE. Further work is needed to evaluate other meta-analytic procedures when the ICC estimates are used as an outcome, like coverage rates for the pooled ICC estimate, the Type I error rates, and the power of Cochran’s *Q* statistic,^79^ and coverage probability of the confidence intervals of $\tau_{\rho }^{2}$ .

There are several limitations to the variance formulas derived for an ICC estimate for reasonable use in meta-analysis of ICC estimates. The formulas derived by Donner and Koval^52^ and Smith^2^ both require knowledge of how many individuals there are per cluster in a primary study. However, typically for CRT studies, primary studies do not report how many individuals there are per cluster. (However, for measurement ICCs, it might be more typical to have the number of measurements per individual.) Also, the $v_{\hat{\rho }}$ formula derived by Hedges et al.^27^ requires knowledge of the variance of the cluster-level variance in a primary study. This is also not often reported in primary studies for either CRT or measurement ICCs, which drastically limits a researcher’s ability to use this formula unless they have access to the primary study’s raw data. Furthermore, some of the formulas require the weighted mean cluster size^2^ ^,^ ^26^ instead of the arithmetic mean cluster size. However, it is not likely that primary CRT design studies will report the weighted mean cluster size, nor be clear about how the average cluster size was calculated if it is reported. Further research is needed to evaluate the use of the mean versus a weighted mean cluster size in the relevant variance formulas.

In summary, we recommend not summarizing across ICCs that come from samples that are too different. The additional obstacle of including ICCs from studies that are capturing variability in outcomes that deviate too much from the primary construct of interest is a further threat to the validity of the resulting inferences due to generalizability error. In addition, researchers are cautioned against use of ICCs when they are based on a sample from a population that is too different than the population that is the focus of the relevant analysis (meta-analysis of effect sizes or power analyses for clustered data).^80^ And the same concern would apply for pooling of measurement ICCs across too heterogeneous populations. However, under the conditions we evaluated, it seems reasonable to use the Fisher TF $v_{\hat{\rho }}$ with RVE or REML to pool ICC estimates, rather than relying on the more ad hoc methods that are typically used for imputing ICC values.

## Data Availability

The R syntax code for this study is openly available in the Open Science Framework at https://osf.io/g5npu.

## Long descriptions

### Figure 1 Long description

A two-panel density plot. Both panels share a y-axis labeled Density ranging from 0.0 to 10.0 and a shared x-axis labeled I C C Estimate ranging from 0.0 to approximately 0.55 on the left and 0.4 on the right.

* Left Panel (English Language Arts): Six colored curves represent grades 3 through 8. Most curves are positively skewed, peaking between I C C values of 0.05 and 0.15. Grade 7 (blue line) shows a sharp primary peak near 0.1 and a smaller secondary peak near 0.28. Grade 8 (pink line) shows multiple small fluctuations extending toward 0.5.

* Right Panel (Mathematics): The distributions are generally broader than the left panel. Grade 5 (green line) has the highest, narrowest peak near 0.08. Grade 8 (pink line) is the flattest, showing a broad distribution that extends further to the right than other grades.

* Legend: Located at the bottom, identifying Grade 3 (red), Grade 4 (olive), Grade 5 (green), Grade 6 (teal), Grade 7 (blue), and Grade 8 (pink).

Navigate back to Figure 1.

### Figure 2 Long description

A multi-panel dot plot with two main sections. The left section represents English Language Arts and the right section represents Mathematics. Each section contains six panels arranged in two rows for Grade 3, 4, 5 (top) and Grade 6, 7, 8 (bottom).

* Axes and Legend: The y-axis is labeled Pooled I C C Estimate, ranging from 0.050 to 0.125. The x-axis within each panel displays six variance I C C methods: Donner (salmon), Fisher (olive), Fisher T F (green), Hedges (teal), Smith (blue), and Swiger (pink). Shapes indicate the pooling method: circles for R E M L and triangles for R V E.

* Left Section (English): In all grades, the data points cluster horizontally between 0.04 and 0.09. A horizontal red dashed line representing the arithmetic mean sits above the data points in most panels, particularly in Grades 4, 7, and 8 where it is positioned near 0.08 or 0.12.

* Right Section (Mathematics): Similar to the left, data points are clustered. In Grade 3, points are near 0.06. In Grades 7 and 8, the points are higher, near 0.08 to 0.10. The red dashed mean line is consistently positioned above the clustered estimates in every grade level.

* Trends: Across both subjects, the R E M L and R V E estimates for each variance method are nearly identical, appearing as overlapping or adjacent shapes. The variance methods (Donner, Fisher, etc.) show very little spread within each grade, indicating high consistency across the different weighting techniques.

Navigate back to Figure 2.

### Table 1 Long description

The table consists of four columns: Total variance, True intraclass correlation, Level-1 variance, and Level-2 variance.

* The Total variance is constant for all rows, defined as sigma squared sub u plus sigma squared sub e equals 100.

* Row 1: rho equals 0.05, sigma squared sub e equals 95, sigma squared sub u equals 5.

* Row 2: rho equals 0.10, sigma squared sub e equals 90, sigma squared sub u equals 10.

* Row 3: rho equals 0.15, sigma squared sub e equals 85, sigma squared sub u equals 15.

* Row 4: rho equals 0.25, sigma squared sub e equals 75, sigma squared sub u equals 25.

* Row 5: rho equals 0.50, sigma squared sub e equals 50, sigma squared sub u equals 50.

* Row 6: rho equals 0.90, sigma squared sub e equals 10, sigma squared sub u equals 90.

Navigate back to Table 1.

### Table 2 Long description

The table consists of two columns: Conditions and Values.

* Row 1: Number of clusters in raw dataset (j). Values are U [30, 50] and U [50, 100].

* Row 2: Average number of units per cluster (n-bar sub j). Values are U [10, 30] and U [30, 50].

* Row 3: Degree of unbalance, zeta. Values are 0.1 and 0.5.

* Row 4: Level-1/Level-2 variance (sigma super 2 sub e / sigma super 2 sub u). Values are 95/5, 90/10, 85/15, 75/25, 50/50, and 10/90.

* Row 5: Number of primary studies (k). Values are 20, 50, and 100.

* Row 6: Between study heterogeneity (tau sub rho). Values are 0.01 and 0.02.

A note at the bottom states: This study assumes one I C C value per primary study.

Navigate back to Table 2.

### Figure 3 Long description

A multi-panel grid with 6 rows and 3 columns.

Axes and Labels:

* The vertical y-axis represents R P B ranging from negative 0.15 to 0.05.

* The horizontal x-axis lists pooling methods: Donner, Fisher, Fisher T F, Hedges, Smith, and Swiger.

* Columns are grouped by k equals 20, 50, and 100 from left to right.

* Rows are grouped by rho values: 0.9, 0.5, 0.25, 0.15, 0.1, and 0.05 from top to bottom.

* Dashed horizontal lines are at y equals 0.05, 0, and negative 0.05.

* Legend at the bottom identifies pink boxes as R E M L and teal boxes as R V E.

Data Trends:

* In the top rows (rho equals 0.9 and 0.5), bias is minimal with most boxplots tightly clustered around the zero line.

* As rho decreases toward 0.05 (bottom row), the R P B values shift downward, indicating an increase in negative bias.

* In the bottom row (rho equals 0.05), most methods show R P B between negative 0.05 and negative 0.15, falling below the acceptable lower boundary.

* The Fisher T F method consistently shows slightly higher R P B (closer to zero) compared to other methods across all panels.

* Increasing k from 20 to 100 results in narrower boxplots, indicating reduced variance in the estimates, though the central bias remains similar across k values for a given rho.

Navigate back to Figure 3.

### Figure 4 Long description

A multi-panel box plot grid with three columns representing k equals 20, 50, and 100, and six rows representing rho values of 0.9, 0.5, 0.25, 0.15, 0.1, and 0.05. The y-axis for all panels is P B ranging from negative 0.015 to 0.005 with a solid horizontal line at 0. The x-axis lists six methods: Donner, Fisher, Fisher T F, Hedges, Smith, and Swiger. Each method has two adjacent box plots: pink for R E M L and teal for R V E.

* In the top row (rho equals 0.9), most methods show P B near 0, except for Fisher T F which shows a slight negative bias.

* In the second row (rho equals 0.5), Fisher T F shows a significant negative bias around negative 0.010, while other methods remain closer to 0 but slightly negative.

* As rho decreases from 0.25 down to 0.05, the P B for most methods stabilizes into a consistent negative bias between negative 0.005 and negative 0.010.

* Fisher T F consistently exhibits different behavior than the other five methods, often showing less negative bias in the lower rho rows compared to its peers.

* The variation across k columns (20, 50, 100) is minimal, suggesting the number of pooled estimates has less impact on P B than the true I C C value.

Navigate back to Figure 4.

### Figure 5 Long description

A multi-panel attribute space diagram organized into two columns and six rows.

Axes and Layout

* The vertical y-axis represents R P B ranging from negative 0.15 to 0.05.

* Horizontal dashed lines are placed at plus or minus 0.05 to indicate acceptable bias levels.

* The horizontal x-axis lists six pooling methods: Donner, Fisher, Fisher T F, Hedges, Smith, and Swiger.

* Columns are categorized by tau sub rho: the left column is 0.01 and the right column is 0.02.

* Rows are categorized by rho values, decreasing from top to bottom: 0.9, 0.5, 0.25, 0.15, 0.1, and 0.05.

Data Trends

* Each method has two adjacent box plots: R E M L in pink and R V E in teal.

* At high rho values (0.9 and 0.5), all methods show very low bias, with box plots tightly clustered around the zero line.

* As rho decreases from 0.25 down to 0.05, the R P B for most methods (Donner, Fisher, Hedges, Smith, Swiger) trends downward, indicating increasing negative bias.

* Fisher T F remains the most stable across all panels, consistently staying closest to the zero line even as rho decreases.

* At the lowest rho value of 0.05, most methods except Fisher T F fall below the negative 0.05 dashed line, indicating unacceptable bias levels.

* The difference between tau sub rho 0.01 and 0.02 is minimal, with similar patterns of bias across both columns.

Navigate back to Figure 5.

### Figure 6 Long description

A multi-panel boxplot grid with 2 columns and 6 rows.

Axes and Legend:

* The Y axis represents P B with a scale from negative 0.015 to 0.005. A solid horizontal line is fixed at 0.

* The X axis lists six pooling methods: Donner, Fisher, Fisher T F, Hedges, Smith, and Swiger.

* The legend at the bottom identifies two estimation methods: R E M L in pink and R V E in light blue.

Grid Structure:

* Columns are categorized by tau sub rho: the left column is 0.01 and the right column is 0.02.

* Rows are categorized by rho values from top to bottom: 0.9, 0.5, 0.25, 0.15, 0.1, and 0.05.

Data Trends:

* At rho equals 0.9, most methods show a slight positive P B above the 0 line, except for Fisher T F which shows a negative P B.

* As rho decreases from 0.9 to 0.05, the boxplots for all methods generally shift downward, indicating an increase in negative P B.

* Fisher T F consistently displays a different distribution pattern compared to the other five methods, often showing a tighter variance or a distinct offset in P B.

* The differences between R E M L and R V E are minimal across most panels, with their boxplots appearing side-by-side with significant overlap.

* The increase in tau sub rho from 0.01 to 0.02 results in slightly wider boxplots, indicating higher variability in the estimates.

Navigate back to Figure 6.

### Figure 7 Long description

The multi-panel graph consists of two columns and six rows.

Column Headers:

* Left column: n bar sub j equals U [10, 30].

* Right column: n bar sub j equals U [30, 50].

Row Labels (Right Side):

* Top to bottom: rho equals 0.9, 0.5, 0.25, 0.15, 0.1, and 0.05.

Axes and Legend:

* Y-axis: R P B ranging from negative 0.15 to 0.05. Dashed horizontal lines are at 0.05 and negative 0.05.

* X-axis: Six pooling methods: Donner, Fisher, Fisher T F, Hedges, Smith, and Swiger.

* Legend: R E M L (pink boxes) and R V E (teal boxes).

Data Trends:

* For high rho values (0.9 and 0.5), the R P B is near zero for all methods, appearing as flat lines.

* As rho decreases toward 0.05, the bias increases (becomes more negative) for most methods, with boxes shifting downward below the negative 0.05 dashed line.

* The Fisher T F method consistently shows the least bias, remaining closer to the zero line than other methods as rho decreases.

* The R E M L and R V E estimates are generally similar within each method, though R V E occasionally shows slightly wider distributions in the lower rho rows.

Navigate back to Figure 7.

### Figure 8 Long description

A multi-panel box plot grid organized into two columns and six rows.

Axes and Legend:

* The Y-axis represents P B, ranging from -0.015 to 0.005 with a solid horizontal line at 0.

* The X-axis lists six pooling methods: Donner, Fisher, Fisher T F, Hedges, Smith, and Swiger.

* The legend at the bottom identifies two estimation methods: R E M L (pink boxes) and R V E (teal boxes).

Column Headers (Average units per cluster):

* Left column: n bar sub j = U [10, 30].

* Right column: n bar sub j = U [30, 50].

Row Labels (True I C C values, rho):

* Rows descend from rho = 0.9 at the top to rho = 0.05 at the bottom.

Data Trends:

* At rho = 0.9 (top row), most methods show a slight positive P B above the 0 line, except Fisher T F which shows a negative P B around -0.005.

* As rho decreases from 0.5 down to 0.05, the P B for most methods (Donner, Fisher, Hedges, Smith, Swiger) shifts from negative values (around -0.005 to -0.010) toward the 0 line.

* Fisher T F exhibits unique behavior: at rho = 0.5, it has the most negative P B (near -0.015), but as rho decreases, its P B increases, becoming the method closest to 0 at rho = 0.05.

* Differences between R E M L and R V E are minimal across all panels, with boxes largely overlapping.

* The right column (larger cluster sizes) generally shows tighter box plots with P B values slightly closer to 0 compared to the left column.

Navigate back to Figure 8.

### Figure 9 Long description

A multi-panel grid with 3 columns and 6 rows. The Y axis represents R M S E ranging from 0.000 to 0.020. The X axis lists six pooling methods: Donner, Fisher, Fisher T F, Hedges, Smith, and Swiger. A dashed horizontal line is at 0.

Columns are grouped by k values: k equals 20, k equals 50, and k equals 100 from left to right. Rows are grouped by rho values: rho equals 0.9, 0.5, 0.25, 0.15, 0.1, and 0.05 from top to bottom.

Each panel contains paired box plots for R E M L (pink) and R V E (teal).

Key observations:

* At rho equals 0.9, R M S E is consistently low across all methods and k values, hovering near 0.005.

* At rho equals 0.5, the Fisher T F method shows a distinct peak in R M S E compared to other methods, which remains relatively stable.

* As rho decreases from 0.25 to 0.05, the R M S E for Fisher T F drops below the other methods, becoming the lowest value in the bottom rows.

* Increasing k from 20 to 100 generally leads to a slight decrease in the spread and median of R M S E across most methods.

* R E M L and R V E values are nearly identical in most panels, appearing as side-by-side boxes of equal height.

Navigate back to Figure 9.

### Figure 10 Long description

A multi-panel box plot grid with two columns and six rows.

Axes and Labels:

- The Y-axis represents R M S E, ranging from 0.000 to 0.020 with a dashed horizontal line at 0.

- The X-axis lists six methods: Donner, Fisher, Fisher T F, Hedges, Smith, and Swiger.

- Columns are categorized by tau sub rho: 0.01 (left) and 0.02 (right).

- Rows are categorized by rho values from top to bottom: 0.9, 0.5, 0.25, 0.15, 0.1, and 0.05.

- Legend at the bottom: Pink boxes represent R E M L and teal boxes represent R V E.

Data Trends:

- In the top row (rho = 0.9), R M S E values are consistently low, near 0.005, across all methods.

- In the second row (rho = 0.5), there is a noticeable spike in R M S E for the Fisher T F method, reaching approximately 0.015, while other methods remain around 0.010.

- As rho decreases from 0.25 down to 0.05, the R M S E for Fisher T F progressively drops, eventually becoming the lowest R M S E value among all methods in the bottom row (rho = 0.05).

- The R E M L and R V E methods show nearly identical performance across all panels, with their box plots overlapping significantly.

- Increasing tau sub rho from 0.01 to 0.02 results in a slight overall upward shift in R M S E values across all panels.

Navigate back to Figure 10.

### Figure 11 Long description

A multi-panel grid with two columns and six rows.

Columns are defined by the average number of units per cluster, n sub j bar. The left column represents n sub j bar equals U [10, 30] and the right column represents n sub j bar equals U [30, 50].

Rows are defined by the true I C C, rho, decreasing from top to bottom: rho equals 0.9, 0.5, 0.25, 0.15, 0.1, and 0.05.

The Y axis for all panels is R M S E, ranging from 0.000 to 0.020. A horizontal dashed line is positioned at 0.000.

The X axis lists six pooling methods: Donner, Fisher, Fisher T F, Hedges, Smith, and Swiger.

Each data point consists of two adjacent box plots: a pink box for R E M L and a teal box for R V E.

Key trends:

- For rho equals 0.9, R M S E is consistently low across all methods, near 0.005.

- For rho equals 0.5 in the left column, Fisher T F shows a distinct spike in R M S E to nearly 0.020, while other methods remain around 0.010.

- As rho decreases, the R M S E for most methods gradually decreases from approximately 0.012 to 0.007.

- The R E M L and R V E estimates are generally similar in height and distribution across most panels.

Navigate back to Figure 11.

### Figure 12 Long description

A multi-panel grid consisting of 18 subplots arranged in 6 rows and 3 columns.

Axes and Legend:

* The Y-axis for all plots is R S E B, ranging from negative 0.10 to 0.10 with increments of 0.05.

* Horizontal dashed lines are positioned at 0.05 and negative 0.05 to indicate acceptable bias boundaries.

* The X-axis labels six pooling methods: Donner, Fisher, Fisher T F, Hedges, Smith, and Swiger.

* Columns are categorized by k values: k equals 20, k equals 50, and k equals 100 from left to right.

* Rows are categorized by rho values: rho equals 0.9, 0.5, 0.25, 0.15, 0.1, and 0.05 from top to bottom.

* A legend at the bottom identifies two pooling types: R E M L represented by pink boxes and R V E represented by teal boxes.

Data Trends:

* Across all panels, the Fisher T F method consistently shows the lowest bias, with box plots centered closest to the zero line.

* As k increases from 20 to 100 (moving left to right), the whiskers of the box plots generally shorten, indicating decreased variance in the estimates.

* As rho decreases from 0.9 to 0.05 (moving top to bottom), the spread of the R S E B values increases, particularly for the Donner, Fisher, Hedges, Smith, and Swiger methods, which often extend beyond the negative 0.05 dashed line.

* R E M L and R V E estimates are paired for each method, showing similar medians but varying degrees of spread depending on the rho and k parameters.

Navigate back to Figure 12.

### Figure 13 Long description

A multi-panel box plot grid organized into two columns and six rows.

Axes and Legend:

* The Y-axis represents R S E B ranging from negative 0.10 to 0.10.

* The X-axis lists six pooling methods: Donner, Fisher, Fisher T F, Hedges, Smith, and Swiger.

* Horizontal dashed lines are set at 0.05 and negative 0.05 to indicate bias boundaries.

* A legend at the bottom identifies two estimation methods: R E M L in pink and R V E in teal.

Grid Structure:

* Columns: The left column represents tau sub rho equals 0.01. The right column represents tau sub rho equals 0.02.

* Rows: From top to bottom, rows represent decreasing rho values: 0.9, 0.5, 0.25, 0.15, 0.1, and 0.05.

Data Trends:

* Most box plots are centered near the zero line, indicating low bias.

* The Fisher T F method consistently shows slightly higher R S E B values compared to other methods across most panels.

* As rho decreases from 0.9 to 0.05 (moving down the rows), the spread of the R V E teal boxes tends to increase relative to the R E M L pink boxes, particularly in the lower rho values where R V E often shows wider interquartile ranges.

* Outliers, represented by grey dots, appear sporadically above and below the whiskers, most notably in the Fisher T F and Swiger categories.

Navigate back to Figure 13.

### Figure 14 Long description

A multi-panel attribute space diagram organized into two columns and six rows.

Vertical Axis: R S E B ranging from minus 0.10 to 0.10. Horizontal dashed lines are placed at 0.05 and minus 0.05 to indicate bias boundaries.

Horizontal Axis: Six pooling methods: Donner, Fisher, Fisher T F, Hedges, Smith, and Swiger.

Columns: The left column represents n sub j bar = U [10, 30] and the right column represents n sub j bar = U [30, 50].

Rows: Organized by rho values from top to bottom: rho = 0.9, 0.5, 0.25, 0.15, 0.1, and 0.05.

Data Representation:

Each method has two adjacent box plots: pink for R E M L and teal for R V E.

- At rho = 0.9, most methods show negative bias, with Fisher T F being the closest to zero.

- As rho decreases from 0.9 to 0.05, the R V E boxes (teal) generally show wider interquartile ranges compared to R E M L (pink), particularly in the lower rho rows.

- For rho = 0.05, the R V E estimates exhibit the highest variability, with whiskers extending toward the minus 0.10 boundary.

- The Fisher T F method consistently maintains a median closest to the zero line across all panels.

- Outliers, represented by grey dots, appear more frequently in the right column (n sub j bar = U [30, 50]) for rho values between 0.15 and 0.9.

Navigate back to Figure 14.

### Figure 15 Long description

A multi-panel figure with two side-by-side box plots. The y-axis is labeled R P B with tick marks at minus 0.05, 0.00, and 0.05. Horizontal dashed lines are drawn at y equals 0.05 and y equals minus 0.05. The x-axis is labeled I C C Parameter with categorical values 0.05, 0.1, 0.15, 0.25, 0.5, and 0.9.

Left Panel: Titled tau sub rho equals 0.01. The box plots show a positive logarithmic trend. At I C C 0.05, the median R P B is approximately minus 0.03. As I C C increases to 0.9, the median R P B rises steadily, ending just below 0.00. All boxes and whiskers remain within the dashed boundary lines.

Right Panel: Titled tau sub rho equals 0.02. The boxes show a similar upward trend but with higher variance and higher overall values. At I C C 0.05, the median is at minus 0.05 with the lower whisker extending below the boundary. At I C C 0.9, the median is at 0.00, and a single outlier dot is visible above the box. The boxes for I C C 0.1 through 0.9 are positioned higher on the y-axis compared to the left panel.

Navigate back to Figure 15.

### Figure 16 Long description

A two-panel boxplot graph. The y-axis is labeled R P B with values from -0.05 to 0.05. The x-axis is labeled I C C Parameter with categorical values 0.05, 0.1, 0.15, 0.25, 0.5, and 0.9. Two horizontal dashed lines are positioned at R P B equals 0.05 and -0.05 to indicate bias boundaries.

Left panel (j equals U [30, 50]):

* At I C C 0.05, the boxplot is centered near -0.05 with high variance.

* As I C C increases to 0.9, the boxes move upward in a logarithmic curve toward the 0.00 line.

* The variance (box height) decreases as I C C increases.

Right panel (j equals U [50, 100]):

* The trend is similar to the left panel but the boxes are more tightly clustered around the median.

* At I C C 0.05, the median is slightly above -0.05.

* At I C C 0.9, the median reaches approximately -0.01.

* Overall, the bias is lower and more stable in the right panel compared to the left panel.

Navigate back to Figure 16.
